# Molecular dissection of Class A PBP function uncovers novel features of the non-canonical *Clostridioides difficile* divisome complex

**DOI:** 10.1371/journal.pgen.1011746

**Published:** 2025-10-21

**Authors:** Gregory A. Harrison, Aimee Shen

**Affiliations:** Department of Molecular Biology and Microbiology, Tufts University School of Medicine, Boston, Massachusetts, United States of America; Texas A&M University, UNITED STATES OF AMERICA

## Abstract

Cell division in bacteria is mediated by the “divisome,” a multiprotein complex that synthesizes the septal peptidoglycan needed to divide one cell into two. We recently showed that the major nosocomial pathogen *Clostridioides difficile* assembles a divisome that is fundamentally distinct from previously studied bacteria because it lacks functional orthologs of the septal peptidoglycan-synthesizing enzymes, FtsW and FtsI. While these enzymes were previously thought to mediate cell division in all walled bacteria, *C. difficile* instead uses the bifunctional Class A Penicillin Binding Protein PBP1 to mediate cell division. Here, we optimized a CRISPRi-based conditional expression system to define features within PBP1 that are critical for its essential functions. Our analyses identify a novel accessory domain that is required for PBP1 function and conserved across Peptostreptococcaceae family PBP1 homologs. We further show that PBP1’s glycosyltransferase and transpeptidase activities are both strictly required for bacterial growth. While PBP1 glycosyltransferase activity is required for septum synthesis during cell division, PBP1’s transpeptidase activity is surprisingly dispensable for cell division, with TPase-deficient (PBP1^TPase*^) cells producing multiple aberrant septa. We demonstrate that the uncontrolled septum synthesis observed in PBP1^TPase*^ cells depends on the non-essential Class B PBP, PBP3, but the catalytic activity of PBP3 is dispensable for this function. Since we also show that PBP3 is recruited to the divisome complex and forms a complex with PBP1, our analyses reveal a cryptic but important regulatory function for PBP3 in promoting *C. difficile* cell division.

## Author summary

Bacterial cell division is an ancient and essential process, but our molecular understanding of this process is primarily based on studies in a select few model systems. Recent work found that the major nosocomial pathogen *Clostridioides difficile* divides by a fundamentally distinct mechanism because homologs of the canonical septum synthesis complex used by virtually all walled bacteria are either missing from the *C. difficile* genome or have lost their function during vegetative division. While this prior work revealed that septum synthesis in *C. difficile* is instead driven by the catalytic activity of the Class A Penicillin Binding Protein called PBP1, the features of PBP1 that enable it to carry out this unusual function were unknown. In the current study, we perform detailed structure-function analyses to determine how *C. difficile* PBP1 has become specialized for this role. We find that PBP1 carries an unusual regulatory domain that is critical for its function. Our analyses also uncover an unexpected function for the non-essential enzyme PBP3 in cell division, identifying a new component of the *C. difficile* cell division complex. These analyses provide new insight into how bacteria can repurpose cell wall synthesis enzymes to fulfill essential functions in novel ways.

## Introduction

The broadly conserved process of bacterial cell division is mediated by a multi-protein complex called the “divisome.” The divisome is comprised of (i) a cytoskeletal scaffold based around the tubulin-like protein FtsZ that organizes the complex in a ring-like structure to mark the site of division and (ii) a transmembrane complex that includes the septal peptidoglycan synthases that drive cytokinesis. Decades of study in model systems have established that the septal peptidoglycan, which ultimately bisects the cell into two daughter cells, is generated by the transmembrane peptidoglycan synthase complex, FtsW-FtsI [1–3]. FtsW is a SEDS family glycosyltransferase that polymerizes the strands of peptidoglycan, and FtsI is a Class B Penicillin Binding Protein (bPBP) that crosslinks strands together through oligopeptide bridges. The activity and localization of the essential FtsW-FtsI complex is further controlled by the FtsQ-FtsL-FtsB transmembrane regulatory sub-complex. This sub-complex guides FtsW-FtsI to the FtsZ-ring at mid-cell and induces the septal PG synthase activity of FtsW-FtsI at the site of division [4–14]. The genes encoding these key divisome proteins are considered universally conserved across bacteria and most can be traced back to the last bacterial common ancestor billions of years ago [15].

While the FtsW-FtsI synthase complex was previously considered universally required for septal peptidoglycan synthesis in all walled bacteria [1,15–17], we recently discovered that *C. difficile* lacks homologs of FtsW and FtsI that function during vegetative cell division [18]. Furthermore, in contrast with previously studied bacteria [8,19–21], the *C. difficile* homologs of the FtsW-FtsI regulators FtsQ, FtsL, and FtsB are dispensable for vegetative cell division [18]. Therefore, *C. difficile* divides without the canonical septal PG synthase complex considered critical for division in virtually all other bacteria.

Since key canonical divisome proteins are either missing from the *C. difficile* genome (FtsW, FtsI) or are dispensable for division (FtsQ, FtsL, FtsB), *C. difficile* must use a unique suite of divisome proteins to carry out cell division [18]. Indeed, in lieu of the canonical FtsW-FtsI PG synthase complex, we recently showed that *C. difficile* uses the bifunctional Class A Penicillin-Binding Protein (**aPBP**) PBP1 to synthesize septal PG [18]. These analyses revealed a novel function for this class of enzymes in driving PG synthesis during cell division, because aPBPs function primarily to reinforce the cell wall by contributing to bulk peptidoglycan biosynthesis in most well-studied bacteria [22,23]. In these systems, aPBPs play a supportive role to the SEDS-bPBP pairs that drive cell elongation (RodA-PBP2) and division (FtsW-FtsI), with aPBPs reinforcing the basement layers of cell wall generated by SEDS-bPBP pairs by filling gaps in the peptidoglycan and repairing damage [24–27]. Notably, in several Bacillota (formerly Firmicutes) species, this function is not essential for growth because aPBPs are completely dispensable for cell viability [28–30].

In contrast to this prevailing model of aPBP function, aPBPs play important roles in driving cell morphogenesis in certain polar-growing bacteria. For instance, Rhizobiales species such as *Agrobacterium tumefaciens* lack RodA-PBP2 altogether and instead rely on aPBP activity to drive cell elongation at cell poles [31,32]. Furthermore, in *Corynebacterium* and *Mycobacterium* species, RodA-PBP2 are dispensable for growth because aPBPs can mediate cell elongation in these organisms [33–36]. Additionally, while aPBPs in *E. coli* move diffusively and pause to synthesize peptidoglycan in a non-directional manner [27,37], in *Streptococcus pneumoniae*, a proportion of PBP1a molecules were recently found to move processively around the mid-cell [38], indicating that aPBPs can exhibit circumferential movement depending on the system. Thus, aPBP function is species-specific.

Our finding that *C. difficile* uses the aPBP PBP1 to drive cell division represents the first example, to our knowledge, of an aPBP driving cell division in the absence of the canonical FtsW-FtsI complex. Yet, how *C. difficile* PBP1 has become specialized for this essential function is unclear. aPBPs in some species harbor accessory domains that contribute to their function at specific sites within the cell. For instance, in *E. coli*, the enzyme PBP1b harbors a UB2H domain, which is recognized by the outer membrane-bound lipoprotein LpoB through gaps in the peptidoglycan mesh [39–42]. LpoB activates PBP1b at these gaps, promoting PBP1b’s ability to fortify sites where the existing peptidoglycan layer is compromised [39,40,42]. While *E. coli* PBP1b is dispensable for growth and division in standard laboratory conditions, it localizes to the site of division and is thought to specifically reinforce the septal peptidoglycan generated by FtsW-FtsI [24,43–46]. Additionally, the *B. subtilis* aPBP PBP1 harbors an extracellular intrinsically disordered region (IDR) that allows it to sense gaps in the peptidoglycan and induce their repair [47], although *B. subtilis* PBP1 is dispensable for growth in standard laboratory conditions [28,48].

In this study, we investigate the function of specific *C. difficile* PBP1 domains by developing a CRISPR-interference (CRISPRi)-compatible trans-complementation system to conditionally express mutant variants of *pbp1* in *C. difficile*. Using this system, we show that the cytosolic and extracellular IDRs of *C. difficile* PBP1 are dispensable for its function and identify a unique, novel regulatory domain of aPBPs that is critical for PBP1 function and conserved in a small subset of bacterial families. By studying the role of PBP1’s catalytic activities, we reveal an unexpected role for the non-essential, monofunctional bPBP, PBP3, in promoting septum synthesis. Collectively, our structure-function analysis of *C. difficile*’s unusual cell division machinery defines key features of *C. difficile*’s essential Class A PBP and provides new insight into PBP function in bacteria.

## Results

### Unique structural features of *C. difficile* PBP1

**Fig 1:**
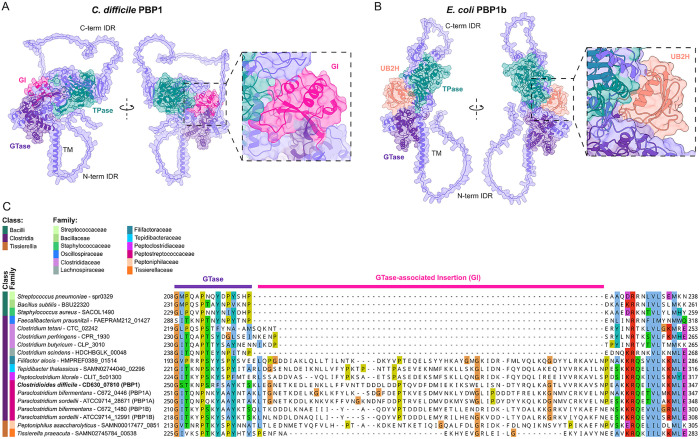
*C*. *difficile* PBP1 harbors an accessory domain conserved in evolutionarily related species as well as N- and C-terminal IDRs. **(A-B)** AlphaFold3 modeling of *C. difficile* PBP1 **(A)** and *E. coli* PBP1B **(B)** shows the C-terminal (C-term) and N-terminal (N-term) IDRs, as well as globular GTase-associated insertion (GI) and UB2H domains. TM indicates the transmembrane helix. **(C)** Protein sequence alignment of PBP1 homologs across Bacillota (formerly Firmicutes) species in the Bacilli, Clostridia, and Tissierellia classes was generated by Clustal Omega and visualized in Jalview. The class and family assigned to each species is based on current assignments in the NCBI taxonomy database [93].

Since *C. difficile* assembles a non-canonical divisome whose activity depends on PBP1, we sought to identify structural features that contribute to PBP1’s unique and essential divisome function. Alphafold3 [49] structural modeling predicts that PBP1 harbors two long intrinsically disordered regions (IDRs) at its cytosolic N-terminus and extracellular C-terminus (Fig 1A). The cytosolic N-terminal IDR is 46 amino acids long, and the extracellular C-terminal IDR is 108 amino acids long.

By comparing the structure of *C. difficile* PBP1 to well-studied PBP1 homologs in Bacillota (formerly Firmicutes) species such as *B. subtilis*, we identified a 66 amino acid domain insertion directly adjacent to *C. difficile* PBP1’s GTase domain that is missing from the PBP1 homologs in *B. subtilis* and other model Bacillota species (Fig 1A). This insertion domain, which we designated as the GTase-associated Insertion (GI) domain, is predicted to form a small globular fold near the linker region that connects the GTase and TPase domains (Fig 1A). Intriguingly, the location of this domain is reminiscent of the UB2H domain of *E. coli* PBP1b (Fig 1B). However, comparison of the GI domain of *C. difficile* PBP1 to the UB2H domain of *E. coli* PBP1b predicts that the GI domain is structurally distinct from the UB2H domain (Fig 1A and 1B).

By running FoldSeek [50] on the predicted structure of the GI domain, we found that this domain was only identified in aPBPs from a select few bacterial families, including the Peptostreptococcaceae, Peptoclostridiaceae, Tepidibacteraceae, Filifactoraceae, and Peptoniphilaceae. One eukaryotic protein was found to harbor a GI domain, an aPBP protein encoded in the genome of the protozoan parasite *Trichomonas vaginalis*. This protein is evolutionarily related to bacterial aPBPs and is encoded in a bacterial gene cassette recently acquired from a *Peptoniphilus* species through horizontal gene transfer [51]. Thus, aside from this instance of interkingdom horizonal gene transfer, the GI domain appears to be unique to bacterial aPBPs within a subset of bacterial families in the Bacillota phylum.

Species within these five bacterial families primarily consist of Gram-positive (monoderm) anaerobic bacteria and occupy a range of anaerobic niches including the gut, oral cavity, vaginal tract, marine mud, and deep-sea hydrothermal vents [52–55]. Notably, species from these bacterial families were formerly classified under the Peptostreptococcaceae [54–56], suggesting that they may have shared features. The Peptostreptococcaceae, Peptoclostridiaceae, Tepidibacteraceae, and Filifactoraceae families are part of the class Clostridia and are primarily comprised of rod-shaped, Gram-positive bacterial species, many of which can form endospores [57]. In contrast, Peptoniphilaceae are non-spore-forming Gram-positive cocci of the class Tissierellia [58]. Aligning PBP1 homologs from representative Bacillota species reveals that the GI domain is unique to this subset of bacterial families and not a general feature of aPBPs across the Bacillota (Fig 1C).

### *pbp1* conditional expression in *C. difficile* using CRISPRi-compatible complementation

**Fig 2:**
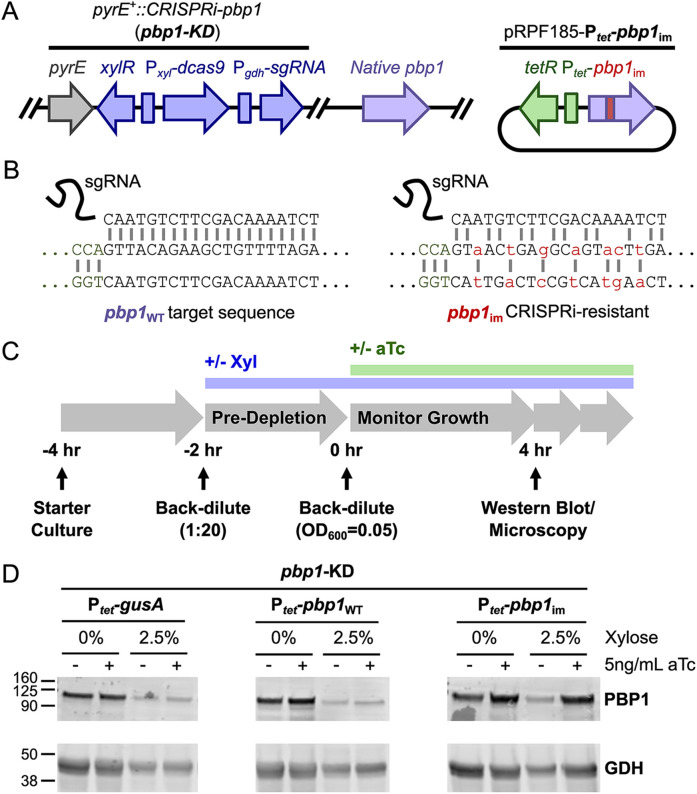
A CRISPRi trans-complementation system that enables the conditional expression of *pbp1* in *C. difficile.* **(A)** Schematic of the *pbp1*-KD strain. A xylose-inducible CRISPRi cassette targeting *pbp1* was inserted downstream of the *pyrE* locus in the *C. difficile* genome. aTc-inducible complementation constructs were supplied on the pRPF185 plasmid [61]. **(B)** To enable complementation of the CRISPRi-KD targeting *pbp1*, synonymous mutations were engineered into the sgRNA-targeting sequence in the plasmid to render *pbp1*_im_ resistant to CRISPRi targeting. **(C)** Schematic of the workflow for depleting PBP1 via the xylose-inducible CRISPRi-KD and rescuing PBP1 levels via the aTc-inducible expression of a CRISPR-immune *pbp1* variant carrying synonymous mutations (B). **(D)** Western blot analysis for PBP1 (96.5 kDa) and loading control GDH (46.0 kDa) using lysate from *pbp1*-KD *C. difficile* complemented with plasmids encoding *P*_*tet*_-*gusA*, -*pbp1*_WT_, or -*pbp1*_im_. PBP1 is depleted during growth in the presence of 2.5% xylose and restored only upon induction of *P*_*tet*_-*pbp1*_im_ with aTc. Blots are representative of three independent experiments, quantification of which can be found in S1A Fig.

We next sought to determine the functional significance of PBP1’s structural features, namely its GI domain and N- and C-terminal IDRs, in regulating PBP1 function in *C. difficile*. To rapidly probe the function of these domains in *C. difficile*, we developed a CRISPR-interference (CRISPRi) trans-complementation method to express conditional alleles of this essential gene. Specifically, we first knocked-down the expression of *pbp1* by integrating a xylose-inducible CRISPRi cassette [59] targeting *pbp1* into a neutral locus downstream of *pyrE* [60] (Fig 2A, left). In the presence of xylose, this *pbp1*-knock down (KD) cassette represses the expression of the endogenous *pbp1* gene. We then complemented the *pbp1*-KD strain with a plasmid expressing CRISPRi-immune *pbp1* variants from an anhydrotetracycline (aTc)-inducible promoter [61] (Fig 2A, right). The aTc-inducible *pbp1* constructs are immune to CRISPRi because they carry synonymous mutations in the sgRNA recognition sequence targeted by the CRISPRi cassette (*pbp1*_im_, Fig 2B). Similar trans-complementation approaches have been used in *Borrelia burgdorferi* and *Mycobacterium tuberculosis* [62,63].

**Fig 3:**
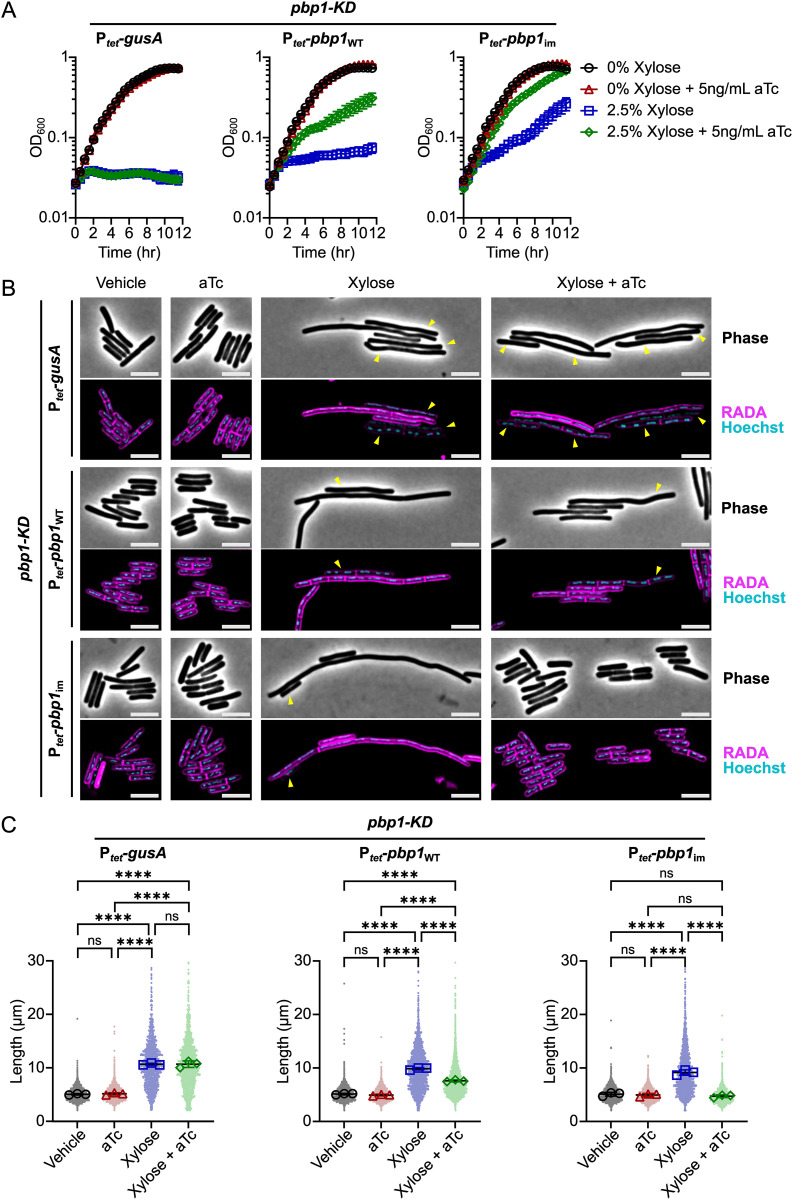
Rescue of *pbp1*-KD phenotypes using CRISPRi trans-complementation. **(A)** Growth of *C. difficile* strains containing the xylose-inducible *pbp1*-KD cassette and aTc-inducible *gusA*, *pbp1*_WT_, or *pbp1*_im_ complementation constructs was monitored by OD_600_. Mean and standard error were calculated across nine biological replicates. **(B)** Fluorescence microscopy analyses of cells exposed to vehicle or 2.5% xylose and/or 5 ng/mL aTc per the scheme in Fig 2C. RADA labeling was used to visualize peptidoglycan synthesis and/or remodeling and Hoechst staining to visualize DNA. Yellow arrows point to a subset of PBP1-depleted cells that have decreased RADA signal, likely due to decreased peptidoglycan metabolism. Quantification of RADA labeling can be found in S2 Fig. Images shown are representative of three independent experiments. Scale bars represent 5 μm. **(C)** Quantification of the length of >1200 cells across three independent experiments. Dots indicate individual cells, and the larger, outlined symbols represent the mean cell length from each replicate. The mean and standard deviation were calculated across replicates; statistical significance was determined by a one-way ANOVA with Tukey’s multiple comparisons test. ns, not significant; **** p < 0.0001.

To verify that our CRISPRi-compatible trans-complementation system complements *pbp1*-KD, we generated *pbp1*-KD *C. difficile* strains carrying plasmids expressing either (i) P_*tet*_-*gusA* (negative control), (ii) P_*tet*_-*pbp1*_WT_, which should remain susceptible to CRISPRi targeting, or (iii) P_*tet*_-*pbp1*_im_, which should be immune to CRISPRi targeting and rescue *pbp1* expression. We pre-depleted PBP1 by culturing each of these strains in the presence of 2.5% xylose for 2 hours to allow the endogenous levels of PBP1 to be diluted through ~2–3 successive division cycles. We then exposed the bacteria to 0 or 5 ng/mL aTc for 4 hours to induce the expression of the complementation construct (Fig 2C). With this experimental set-up, the bacteria experienced *pbp1-*KD for 6 hours (2 hours pre-depletion followed by 4 additional hours of treatment) and induction of the immune construct for 4 hours, all while maintaining the cultures in logarithmic growth (Fig 2C). Exposure to xylose significantly decreased PBP1 levels, while aTc addition increased PBP1 levels in the P_*tet*_-*pbp1*_im_ strain but not the negative control strains (Fig 2D, S1A Fig). Notably, while the *pbp1-*KD did not fully deplete PBP1 (Fig 2D, S1A Fig), its levels were sufficiently reduced such that the growth inhibition and filamentation phenotypes associated with PBP1 depletion were observed (Fig 3) [18,59]. Importantly, induction of the CRISPRi-immune *pbp1*_im_ using 5 ng/mL aTc almost fully complemented the growth inhibition caused by *pbp1*-KD, in contrast with the negative controls (*gusA* or *pbp1*_WT_, Fig 3A, green diamonds). It should also be noted that expression from the aTc-inducible promoter is leaky, such that even in the absence of aTc addition, we observed a modest restoration of growth in cells harboring P_*tet*_*-pbp1*_im_ (Fig 3A; blue squares). Taken together, these analyses demonstrate that a CRISPRi trans-complementation system can be used to effectively knock-down native *pbp1* expression and heterologously express an ectopic, CRISPRi-resistant variant of *pbp1*.

Since *pbp1*-KD also causes cell filamentation due to defects in cell division [18,59] (Fig 3B), we examined the CRISPRi trans-complementation strains using microscopy. Specifically, we used the fluorescent D-amino acid RADA to visualize sites of active peptidoglycan metabolism or remodeling and Hoechst to stain the bacterial nucleoid. As expected, *pbp1*-KD caused cell filamentation, although occasional septa could be detected within filaments, similar to previously published results [18,59]. Conditional expression of *pbp1*_im_ restored normal cell morphology and cell length (Fig 3B and 3C). PBP1 depletion also induced the formation of a population of cells with decreased RADA signal likely due to decreased peptidoglycan metabolism (Fig 3B, yellow arrows) (S2 Fig), which is reversed upon conditional expression of *pbp1*_im_ (S2 Fig). While induction of *pbp1*_WT_ partially restored the growth and cell length phenotypes caused by *pbp1*-KD (Fig 3), no change in PBP1 protein levels was detected (Fig 2D, S1A Fig) likely because western blots have limited sensitivity in quantifying small changes in protein levels. Regardless, our analyses show that *pbp1*_im_ induction successfully complements the phenotypes of CRISPRi *pbp1*-KD.

We additionally noted that simply over-expressing *pbp1* caused no change in cell length (Fig 3C), although a subtle but significant increase in cell width from ~0.8 μm to ~0.9 μm was observed (S1B Fig). Increased bacterial cell width upon over-production of aPBP enzymes has previously been observed in *B. subtilis*, and to a lesser extent in *E. coli* [25,64]. To examine if PBP1 levels correlated with *C. difficile* cell width, we titrated PBP1 induction with aTc (5 ng/mL to 50 ng/mL). We found that over-production of PBP1 caused a dose-dependent increase in cell width, with no change in cell length (S3 Fig). This finding indicates that PBP1 activity likely impacts *C. difficile* morphology beyond septum synthesis. Indeed, PBP1 is also localized to the sidewall [18,65], and may play a role in both sidewall and septum synthesis in *C. difficile*.

### PBP1’s IDRs are not strictly required for growth or division in *C. difficile*

**Fig 4:**
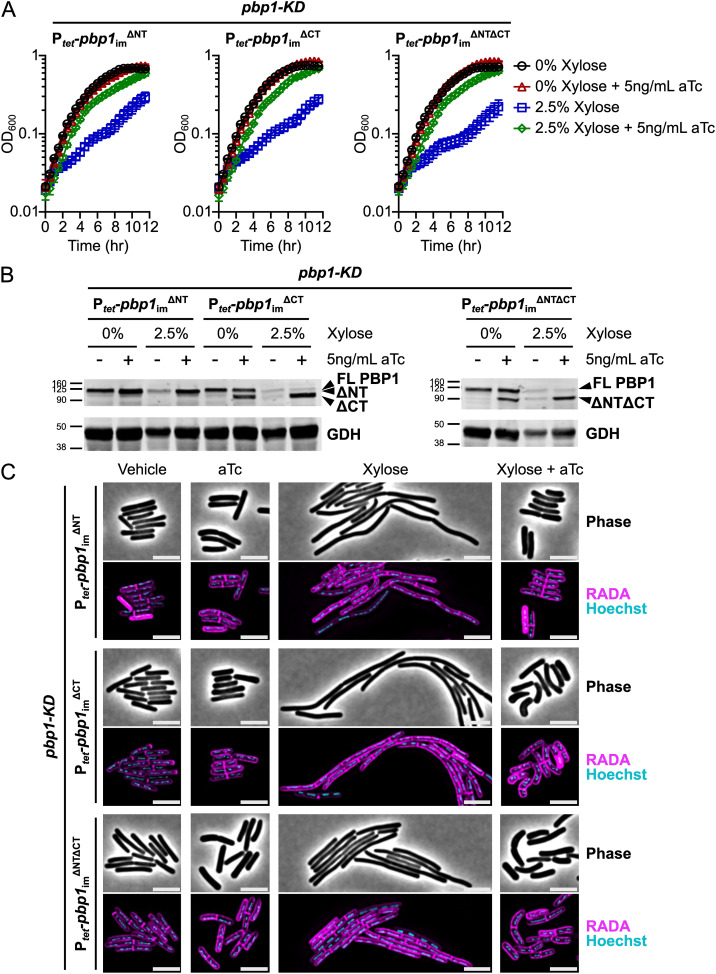
PBP1 N-terminal and C-terminal IDRs are not strictly required for growth or division. **(A)** Growth of *C. difficile* strains containing the xylose-inducible *pbp1*-KD cassette and aTc-inducible *pbp1*_im_^ΔNT^, *pbp1*_im_^ΔCT^, or *pbp1*_im_^ΔNTΔCT^ complementation constructs was monitored by OD_600_. Mean and standard error were calculated across nine biological replicates. **(B)** Western blot analyses of PBP1 in cells exposed to vehicle or 2.5% xylose and/or 5 ng/mL aTc per the scheme in Fig 2C. GDH (46.0 kDa) was used as a load control. FL indicates Full-Length PBP1, and truncated protein variants are indicated. Blots are representative of three independent experiments, and their quantification is presented in S4A Fig. **(C)** Fluorescence microscopy analyses of cells exposed to vehicle or 2.5% xylose and/or 5 ng/mL aTc per the scheme in Fig 2C. RADA labeling was used to visualize peptidoglycan synthesis and/or remodeling and Hoechst staining to visualize DNA. Images shown are representative of three independent experiments. Scale bars represent 5 μm. Cell length quantification across replicates is presented in S4B Fig.

Having developed a CRISPRi trans-complementation system to express mutant variants of *pbp1*_im_, we next tested the function of mutant *pbp1*_im_ alleles. Notably, our system enables *pbp1* mutant alleles to be rapidly tested within 2–3 days compared to the multiple weeks required to assess the function of chromosomally-encoded mutant alleles. We first examined the functional requirements for the N-terminal cytosolic IDR and C-terminal extracellular IDR by introducing plasmids encoding PBP1 variants lacking the 46-amino acid N-terminal IDR (P_*tet*_-*pbp1*_im_^ΔNT^), the 108-amino acid C-terminal IDR (P_*tet*_-*pbp1*_im_^ΔCT^), or both IDRs (P_*tet*_-*pbp1*_im_^ΔNTΔCT^) into the *pbp1*-KD strain. Following xylose-inducible PBP1 pre-depletion and aTc-inducible trans-complementation (Fig 2C), we found that conditional expression of *pbp1*_im_^ΔNT^, *pbp1*_im_^ΔCT^, or *pbp1*_im_^ΔNTΔCT^ rescued the growth inhibition caused by *pbp1*-KD as measured by OD_600_ over time (Fig 4A). Thus, neither the N-terminal nor the C-terminal IDR are required for PBP1 to support the growth of *C. difficile* in standard laboratory conditions. Importantly, all three of these truncated protein variants were stably produced during knock-down of the native *pbp1* (Fig 4B, S4A Fig). Conditional expression of *pbp1*_im_^ΔNT^, *pbp1*_im_^ΔCT^, or *pbp1*_im_^ΔNTΔCT^ all restored WT cell length (Fig 4C, S4B Fig), indicating that the N- and C-terminal IDRs are dispensable for PBP1 function during *C. difficile* cell division. Conditional expression of both *pbp1*_im_^ΔCT^ and *pbp1*_im_^ΔNTΔCT^ modestly increased cell curvature, with comma-shaped cells frequently observed in these two mutants (Fig 4C). While the overall mean cell curvature of the cells did not increase in a statistically significant manner, there was a small but significant increase in the frequency of curved cells in the population (S5 Fig). Thus, our data show that PBP1’s IDRs are not strictly required for growth or division in standard laboratory conditions, but the extracellular C-terminal IDR may play a minor role in promoting normal cell morphology.

### The GI domain in PBP1 is critical for its function in *C. difficile*

**Fig 5:**
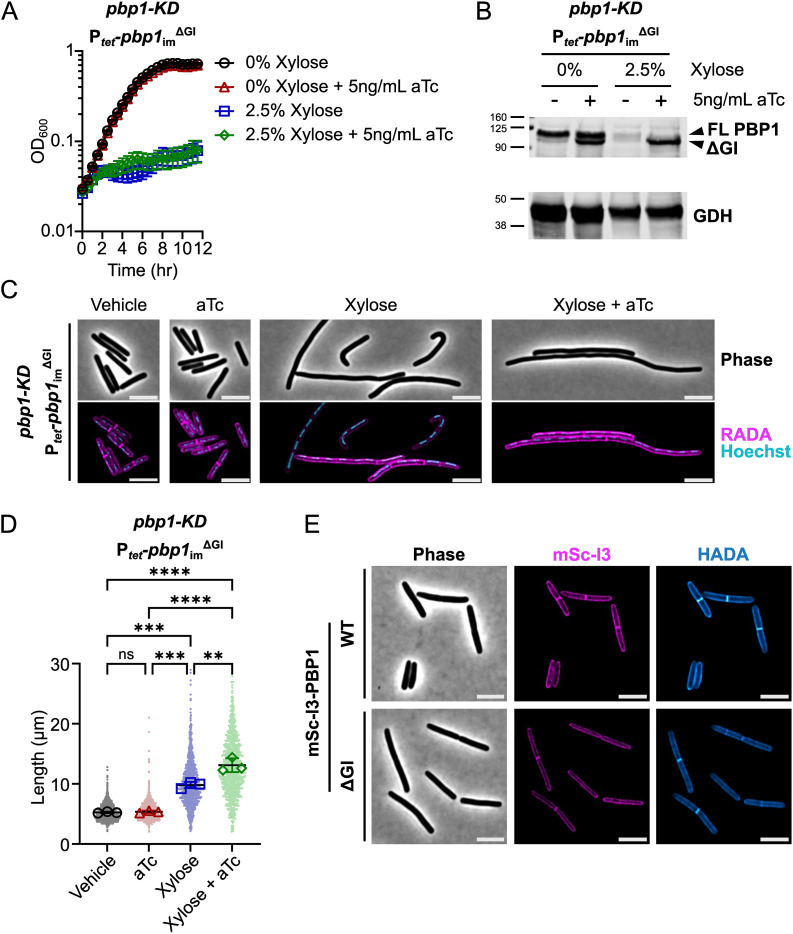
The GI domain of PBP1 is critical for *C. difficile* growth and division. **(A)** Growth of the *C. difficile* strain containing the xylose-inducible *pbp1*-KD cassette and an aTc-inducible *pbp1*_im_^ΔGI^ complementation construct was monitored by OD_600_. Mean and standard error were calculated across nine biological replicates. **(B)** Western blot analyses of PBP1 in cells exposed to vehicle or 2.5% xylose and/or 5 ng/mL aTc per the scheme in Fig 2C. GDH (46.0 kDa) was used as a load control. FL indicates full-length PBP1, and the band representing the ΔGI variant is indicated. Blots are representative of three independent experiments, and quantification across experiments is presented in S7 Fig. **(C)** Fluorescence microscopy of cells exposed to 2.5% xylose and/or 5 ng/mL aTc per the scheme in Fig 2C. RADA labeling was used to visualize peptidoglycan synthesis and/or remodeling and Hoechst staining to visualize DNA. Images are representative of three independent experiments. **(D)** Quantification of cell length of >900 cells across three independent experiments. Dots indicate individual cells. The larger, outlined symbols represent the mean cell length from each replicate. The mean and standard deviation were calculated across replicates; statistical significance was determined between replicates by a one-way ANOVA with Tukey’s multiple comparisons test. ns, not significant; ** p < 0.01, *** p < 0.001, **** p < 0.0001. **(E)** Localization of mScI3-PBP1 produced under the control of an aTc-inducible promoter using fluorescence microscopy. Cells were cultured in the presence of 5 ng/mL aTc for 1 hr, exposed to HADA for 10 min to label peptidoglycan synthesis and/or remodeling, and then fixed for microscopy. Scale bars represent 5 μm.

We next assessed the functional significance of the other major structural feature in *C. difficile* PBP1 predicted by structural modeling, the GI domain, by introducing a plasmid encoding a GI domain mutant (PBP1^ΔGI^) into the *pbp1*-KD strain. The deletion construct lacks residues 267–328 and was designed based on AlphaFold3 modeling, which predicts that the functional domains of PBP1^ΔGI^ adopt a similar structure as in PBP1^WT^ (S6A and S6B Fig). Trans-complementation of the *pbp1*-KD with the aTc-inducible *pbp1*_im_^ΔGI^ construct revealed that loss of the GI domain prevented PBP1 function, as measured by bacterial growth (Fig 5A). Importantly, PBP1^ΔGI^ was stably produced during our induction conditions based on western blot analyses (Fig 5B, S7 Fig), indicating that the GI domain is critical for the proper functioning of PBP1.

Consistent with the observed growth defects, *pbp1*_im_^ΔGI^ expression failed to reverse the filamentation phenotype caused by *pbp1*-KD, and even exacerbated its filamentation phenotype (Fig 5C and 5D). Thus, the GI domain is required for PBP1 to synthesize septal peptidoglycan. To determine if the GI domain impacts the localization of PBP1 to sites of septal peptidoglycan synthesis, we analyzed the localization of PBP1 and PBP1^ΔGI^ variants with N-terminal fusions to mScarlet-I3 (mSc-I3) [66] in a WT background. The fusion protein constructs were integrated into an ectopic site in the chromosome, generating merodiploid strains that also express the native copy of *pbp1*. mSc-I3-PBP1^ΔGI^ localized to both the sidewall and at septa, similar to the mSc-I3-PBP1^WT^ [18] (Fig 5E). Thus, the GI domain is not required for proper protein localization and is instead likely required for the activity of PBP1 at the sites of peptidoglycan synthesis.

### PBP1 GTase and TPase activities are essential for growth

While these data reveal that the GI domain promotes PBP1 function, it is unclear whether PBP1’s distinct catalytic activities are specifically required during *C. difficile* growth and/or division. Given that inhibition of aPBP GTase activity with Moenomycin A (MoeA) recapitulates the effects of *pbp1*-KD [18], PBP1’s GTase activity is likely essential for its function in *C. difficile*. However, whether PBP1’s TPase activity is essential for its function remains unclear. Prior studies have proposed that PBP1’s TPase activity is dispensable for its function because β-lactam antibiotics that potently inhibit PBP1 TPase activity *in vitro* do not necessarily inhibit *C. difficile* growth [67]. Furthermore, the TPase activity of the essential division-specific bPBP FtsI is dispensable in both *B. subtilis* and *S. aureus* because other bPBPs can compensate for the loss of this activity [3,68]. In these species, the essential function of FtsI is to allosterically activate FtsW, while FtsI’s TPase activity can be supplied by functionally redundant TPases [3,68]. We therefore tested whether *C. difficile* PBP1’s GTase or TPase catalytic activities are essential for its function using the CRISPRi conditional expression system.

To abrogate PBP1’s GTase activity, we generated a *pbp1*_im_ construct encoding two amino acid substitutions in the active site of the GTase domain within the conserved EDxxFxxHxG motif, E137Q/D138N (*pbp1*_im_^GTase*^). This mutation includes the catalytic glutamate residue and the adjacent aspartate, which is thought to play a key role in the catalytic mechanism [69]. We separately inactivated PBP1 TPase activity by generating a *pbp1*_im_ construct encoding a single amino acid change in the nucleophilic serine residue in the conserved SxxK motif within the TPase active site, S487A (*pbp1*_im_^TPase*^).

**Fig 6:**
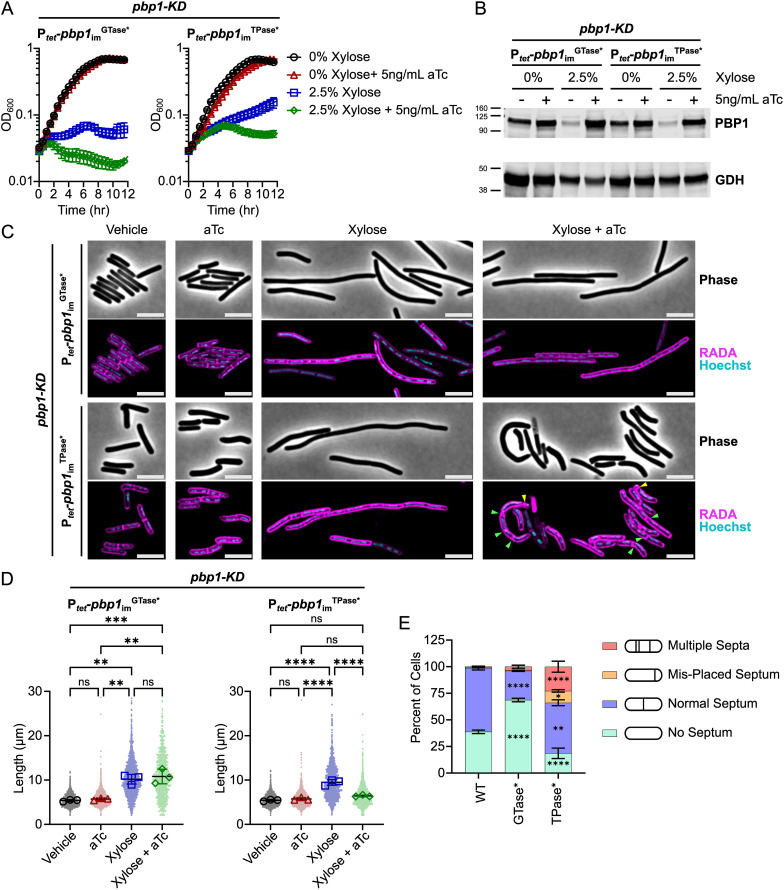
Both the GTase and TPase activities of PBP1 are essential for *C. difficile* growth, but PBP1 TPase-deficient cells can still form septa and complete cell division. **(A)** Growth of *C. difficile* strains containing the xylose-inducible *pbp1*-KD cassette and aTc-inducible *pbp1*_im_^GTase*^ or *pbp1*_im_^TPase*^ complementation constructs was monitored by OD_600_. Mean and standard error were calculated across nine biological replicates. **(B)** Western blot analyses of PBP1 in cells exposed to vehicle or 2.5% xylose and/or 5 ng/mL aTc per the scheme in Fig 2C. GDH (46.0 kDa) was used as a load control. Blots are representative of three independent experiments, and quantification across experiments is presented in S8 Fig. **(C)** Fluorescence microscopy analyses of cells exposed to 2.5% xylose and/or 5 ng/mL aTc per the scheme in Fig 2C. RADA labeling was used to visualize peptidoglycan synthesis and/or remodeling and Hoechst staining to visualize DNA. Images are representative of three independent experiments. Yellow arrows point to polar septa being formed, and green arrows point to instances of aberrant puncta near septa, bulky septa, and/or multi-septate cells. Scale bars represent 5 μm. **(D)** Quantification of cell lengths for >800 cells across three independent experiments. Dots indicate individual cells, and the larger, outlined symbols represent the mean cell length from each replicate. The mean and standard deviation were calculated across replicates. **(E)** Quantification of septum labeling phenotypes for >500 bacteria across three independent experiments. The mean as a percent of total cells was calculated across replicates, and error bars indicate standard error of the mean. In panels E and D, statistical significance was determined between replicates by a one-way ANOVA with Tukey’s multiple comparisons test (D) or a two-way ANOVA with Holm-Šídák’s multiple comparisons test **(E)**. ns, not significant; * p < 0.05, ** p < 0.01, *** p < 0.001, **** p < 0.0001.

Conditional expression of *pbp1*_im_^GTase*^ or *pbp1*_im_^TPase*^ failed to complement the growth defect caused by *pbp1*-KD (Fig 6A), indicating that the GTase and TPase activity of PBP1 are essential for *C. difficile* growth. These data further indicate that other TPases encoded by *C. difficile*, namely the two bPBPs and five L, D-TPases (LDTs) [18,70,71], cannot support the growth of *C. difficile* in the absence of PBP1 TPase activity. Notably, both PBP1^GTase*^ and PBP1^TPase*^ were stably expressed in *C. difficile* as measured by western blot (Fig 6B, S8 Fig).

### PBP1 TPase-deficient cells complete cell division but exhibit aberrant PG incorporation and uncontrolled septum synthesis

We next examined the morphology of conditional expression strains where PBP1’s GTase or TPase activities were genetically inactivated. Consistent with prior analyses showing that the chemical inhibition of PBP1’s GTase activity with MoeA inhibits cell division [18], *C. difficile* cells conditionally expressing *pbp1*_im_^GTase*^ exhibited a filamentous morphology and thus decreased septum synthesis (Fig 6C and 6D). In contrast, *C. difficile* cells conditionally expressing *pbp1*_im_^TPase*^ had an overall cell length similar to vehicle-treated control cells, indicating that PBP1 transpeptidase activity is not strictly required for completing cell division. However, *pbp1*_im_^TPase*^ cells appeared to exhibit dysregulated cell wall synthesis because we observed greater variability in cell length (Fig 6C and 6D), and PBP1 TPase-deficient cells more frequently exhibited multiple septa or septa placed near the cell poles (Fig 6C and 6E) and increased aberrant RADA puncta observed per cell (S9 Fig), compared to *pbp1*_im_ control or *pbp1*_im_^GTase*^-expressing cells. Taken together, our data indicate that *C. difficile* cells conditionally expressing *pbp1*_im_^TPase*^ complete septum synthesis in an uncontrolled manner, generating irregular septa with abnormal RADA labeling.

### PBP3 promotes aberrant PG incorporation and abnormal septum synthesis in PBP1 TPase-deficient cells in a non-catalytic manner

Our finding that *pbp1*_im_^TPase*^ cells still generate septa and divide to maintain a population of cells with predominantly normal cell lengths suggests that another enzyme could provide the TPase activity during septum synthesis. We further hypothesized that this additional TPase might drive the uncontrolled synthesis of septa and aberrant puncta of RADA incorporation observed in these cells. *C. difficile* encodes three bPBPs with TPase activity: the sporulation-specific bPBP, SpoVD, which pairs with the SEDS enzyme SpoVE [18,72] and is essential for sporulation; the sidewall-synthesis enzyme PBP2, which pairs with the SEDS enzyme RodA [18]; and an orphan bPBP, PBP3, which is induced during sporulation but has only subtle effects on sporulation and vegetative growth [18,73]. SpoVD functions exclusively during sporulation and is undetectable in vegetative cells [18,72]. While PBP2 was recently found to localize to the site of division in a proportion of cells [65], knock-down studies suggest that PBP2 functions primarily during cell elongation [18]. Thus, we hypothesized that PBP3 could promote uncontrolled septum synthesis in cells conditionally expressing *pbp1*_im_^TPase*^. This hypothesis was based on the following observations: (i) PBP3 promotes asymmetric division during sporulation when SpoVD catalytic activity is inactivated, indicating a role in the sporulation-associated division complex [73], and (ii) loss of *pbp3* leads to a statistically significant increase in *C. difficile* cell length (~25%) during vegetative growth [18]. These findings imply that PBP3 is present at low levels during vegetative growth and that it modulates *C. difficile* vegetative cell division, which could be similar to its role in promoting asymmetric division during sporulation.

**Fig 7:**
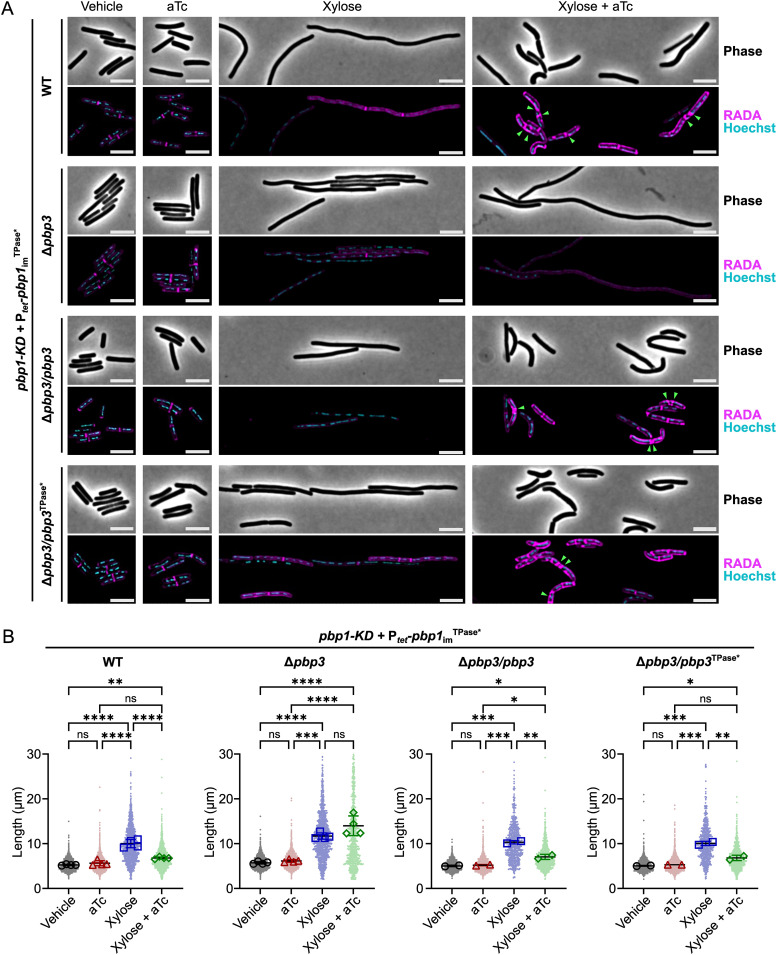
PBP3 promotes uncontrolled septum synthesis and aberrant peptidoglycan incorporation in PBP1 TPase-deficient cells. **(A)** Growth of *C. difficile* strains containing the xylose-inducible *pbp1*-KD cassette in WT or a Δ*pbp3* mutant and a plasmid-encoded, aTc-inducible *pbp1*_im_^TPase*^ complementation construct. Complementation of Δ*pbp3* was accomplished by expressing *pbp3* or *pbp3*^TPase*^ as a bicistronic construct downstream of *pbp1*_im_^TPase*^ under the P_*tet*_ promoter. Cells exposed to 2.5% xylose and/or 5 ng/mL aTc per the scheme in Fig 2C were subject to RADA labeling for 10 min to visualize peptidoglycan synthesis and/or remodeling and Hoechst staining for DNA. Images are representative of 2-4 independent experiments. Green arrows indicate examples of irregular septa being formed. Scale bars represent 5 μm. **(B)** Quantification of cell lengths for >600 cells across 2-4 independent experiments. Dots indicate individual cells, and the larger, outlined symbols represent the mean cell length from each replicate. The mean and standard deviation were calculated across replicates; statistical significance was determined between replicates by a one-way ANOVA with Tukey’s multiple comparisons test. ns, not significant; * p < 0.05, ** p < 0.01, *** p < 0.001, **** p < 0.0001.

To test whether PBP3 contributes to uncontrolled septum synthesis in PBP1 TPase-deficient cells, we conditionally expressed the *pbp1*_im_^TPase*^ construct in a mutant lacking *pbp3*. Conditionally expressing the *pbp1*_im_^TPase*^ construct in the absence of PBP3 induced severe filamentation and decreased overall RADA incorporation, similar to cells depleted of PBP1 (*pbp1*-KDΔ*pbp3*/P_*tet*_-*pbp1*_im_^TPase*^ vs. *pbp1*-KD/P_*tet*_-*pbp1*_im_^TPase*^, Fig 7, S10 Fig). These data indicate that the formation of septa and the aberrant peptidoglycan insertion observed in PBP1^TPase*^-producing cells relies on the presence of PBP3. Consistent with this conclusion, complementing back the expression of *pbp3* in the *pbp1*-KDΔ*pbp3*/P_*tet*_-*pbp1*_im_^TPase*^ strain prevented filament formation and restored the cell length phenotype of the WT background conditionally expressing *pbp1*_im_^TPase*^ (Fig 7). Complementation also induced the uncontrolled septum formation and aberrant RADA incorporation that distinguishes the *pbp1*_im_^TPase*^ strain (Fig 7). Notably, western blot analyses confirmed that PBP1^TPase*^ is stably expressed in the Δ*pbp3* background (S11 Fig), indicating that PBP3 does not impact PBP1^TPase*^ stability but is instead required for triggering septum formation in the PBP1^TPase*^-producing cells.

Since functionally redundant bPBPs can compensate for the loss of the catalytic activity of the FtsI bPBP in some bacteria [3,68], we tested whether the TPase activity of PBP3 was necessary for the *pbp1*_im_^TPase*^ strain to complete cell division. To this end, we complemented the Δ*pbp3* strain conditionally expressing *pbp1*_im_^TPase*^ (*pbp1*-KDΔ*pbp3*/P_*tet*_-*pbp1*_im_^TPase*^) with a construct encoding a TPase catalytic mutant of PBP3. This construct encodes a single amino acid substitution in the conserved catalytic SxxK motif of PBP3’s active site, S299A (*pbp3*^TPase*^). Surprisingly, the *pbp3*^TPase*^ complementation construct was sufficient to restore uncontrolled septum formation in Δ*pbp3* cells conditionally expressing the *pbp1*_im_^TPase*^ allele (Fig 7). Thus, PBP3 TPase activity is not required to promote septum synthesis and cell division in cells conditionally producing PBP1^TPase*^. Rather, the presence of PBP3 is needed to induce uncontrolled septum synthesis in PBP1 TPase-deficient cells.

### PBP3 is a component of the *C. difficile* divisome complex

**Fig 8:**
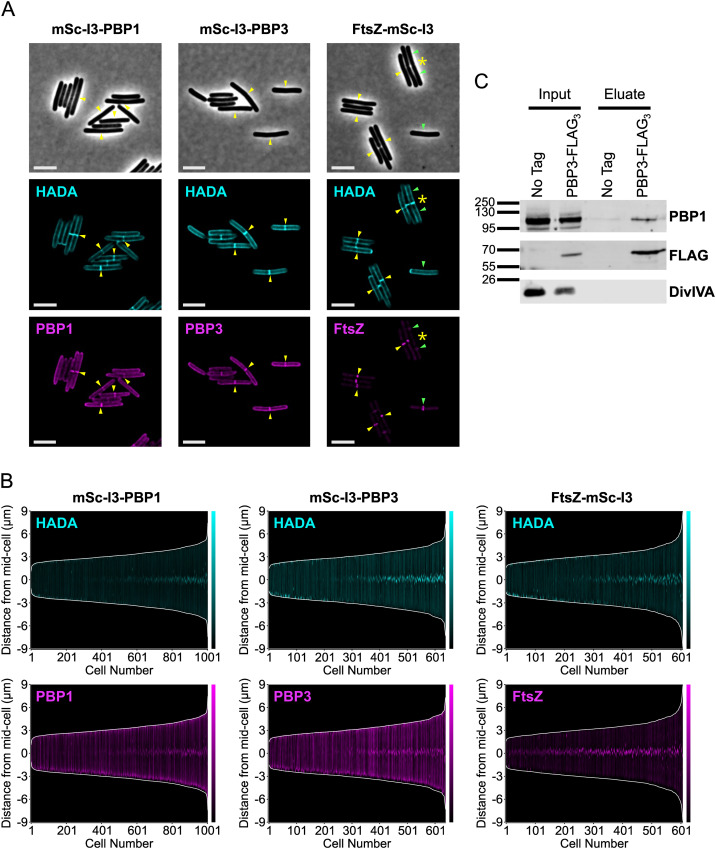
PBP3 can localize to the *C. difficile* divisome and forms a complex with PBP1. **(A)** Localization of fluorescently-tagged proteins in *C. difficile* carrying either an N-terminal (PBP1 and PBP3) or C-terminal (FtsZ) mSc-I3 tag under the control of an aTc-inducible promoter. Cells were cultured in the presence of either 5 ng/mL aTc (mSc-I3-PBP1 and mSc-I3-PBP3) or 0.5 ng/mL aTc (FtsZ-mSc-I3) for 1 hr, exposed to HADA for 10 min to label peptidoglycan synthesis and/or remodeling, and then fixed for microscopy. Yellow arrows indicate co-localization of mSc-I3 signal and HADA. For FtsZ-mSc-I3, septa with no mSc-I3 signal are indicated with a yellow asterisk (*); these represent late-stage septa with no FtsZ localization. Green arrows indicate that the mSc-I3 signal does not co-localize with noticeable HADA incorporation; these likely represent early FtsZ-rings that have not yet initiated septum synthesis. Scale bars represent 5 μm. **(B)** Demographs depicting the medial axis fluorescence profile of >600 cells analyzed via MicrobeJ. Each cell is indicated on the x-axis, and cells are ordered by length. The cell length is indicated by the white lines, whereas the HADA or mSc-I3 signal is depicted in cyan or magenta, respectively. The HADA signal intensity was normalized across all images and demographs to facilitate direct comparisons between samples, whereas the mSc-I3 signal was scaled independently for each individual reporter construct. **(C)** Co-immunoprecipitation analysis of PBP3-FLAG_3_. Δ*pbp3* strains were engineered to harbor a P_*xyl*_-*pbp3*-*FLAG*_3_ construct or a *P*_*xyl*_-*pbp3* no-tag control. Cultures were grown in the presence of 0.5% xylose, then cells were lysed, membrane proteins were solubilized with 0.5% DDM, and anti-FLAG resin was used to immunoprecipitate the bait protein. PBP3-FLAG_3_, PBP1, and the negative control DivIVA were detected by western blotting.

Since a possible explanation for these data is that PBP3 plays a regulatory role in activating septum synthesis, perhaps by enhancing the GTase activity of PBP1^TPase*^ and ultimately causing dysregulation of peptidoglycan synthesis in these cells, we tested whether PBP3 is recruited to the vegetative *C. difficile* divisome. Specifically, we compared the localization profile of PBP3 to those of known divisome proteins, PBP1 and FtsZ [18,74], using mSc-I3 protein fusions. Constructs encoding the fusion proteins inducibly expressed from the P_*tet*_ promoter were integrated downstream of the *pyrE* locus in WT *C. difficile*. After transiently inducing the expression of the fusion protein constructs with aTc and visualizing *de novo* peptidoglycan synthesis with HADA, we found that PBP3 localized both to septa and the sidewall, similar to PBP1 (Fig 8A). In demographs comparing mSc-I3 and HADA labeling across the medial axis of cells, PBP1 and PBP3 recruitment to mid-cell is coincident with the onset of septum synthesis, as indicated by HADA incorporation at mid-cell (Fig 8B). By comparison, FtsZ localization was restricted to the mid-cell, and this localization was observed prior to the onset of septum synthesis (Fig 8A and 8B). These data indicate that PBP3 can localize to the site of septum synthesis and the sidewall, similar to PBP1, and therefore may play a role both in vegetative cell division as part of the *C. difficile* divisome as well as in sidewall synthesis.

We next assessed whether PBP3 interacts with PBP1 during vegetative cell growth. Notably, we previously reported that *C. difficile* PBP1 and PBP3 interact in a bacterial two-hybrid assay performed in *E. coli* and this interaction is also observed during sporulation [73]. To determine if PBP1 and PBP3 also interact during vegetative growth in *C. difficile*, we generated strains that inducibly express constructs encoding either PBP3 or PBP3-FLAG_3_ (prior RNA-Seq analyses indicated that *pbp3* is expressed at low levels in vegetative cells [75]). Actively growing cultures of the resulting strains were treated with formaldehyde crosslinker to stabilize protein-protein interactions, lysed, and the membrane proteins were solubilized with n-dodecyl-β-D-maltoside before performing the anti-FLAG co-immunoprecipitation. PBP3-FLAG_3_ co-immunoprecipitated with PBP1 but not with the negative control DivIVA, a membrane-associated protein that is also present at the site of division [76], suggesting that PBP3 and PBP1 are able to interact either directly or indirectly in vegetatively growing *C. difficile* (Fig 8C). Collectively, our findings indicate that PBP3 and PBP1 localize to the site of septum synthesis in dividing vegetative *C. difficile* and that these two proteins interact, directly or indirectly, as part of the same complex. Based on these observations, we conclude that PBP3 is an accessory component of the *C. difficile* divisome complex that functions to promote its septum synthesis activity.

## Discussion

By taking advantage of newly developed genetic tools in *C. difficile* to establish a CRISPRi-based trans-complementation system, we performed structure-function analyses of PBP1, the essential peptidoglycan synthase required for cell growth and division [18,59]. This system allowed us to rapidly determine the requirement for discrete domains within PBP1 and its enzymatic activities, which would have been difficult to accomplish by engineering mutations in the native *pbp1* locus within the *C. difficile* genome.

Many Bacillota species have aPBPs with extracellular C-terminal IDRs. The precise role of these IDRs is not fully understood, but there is recent data suggesting they are important. In *B. subtilis*, the extracellular IDR of PBP1 was found to promote its activity particularly during cell wall stress [47]. The IDR of *B. subtilis* PBP1 is thought to sense gaps in the peptidoglycan that are then repaired by PBP1. Recently, the extracellular IDR of PBP1a in multiple streptococcal species, including *S. pneumoniae*, *S. mutans*, and *S. pyogenes,* was found to be *O*-glycosylated [77]. This modification appears to prevent proteolytic degradation, thereby regulating the levels and stability of this key aPBP [77]. We found that PBP1’s N- and C-terminal IDRs are both dispensable for *C. difficile* growth and division. We nevertheless observed slight increases in the frequency of curved cells in PBP1^ΔCT^- and PBP1^ΔNTΔCT^-expressing cells (S5 Fig), which could be consistent with uneven deposition of peptidoglycan on the sidewall [47,78]. While these data suggest that the extracellular C-terminal IDR of *C. difficile* PBP1 plays a minor role in cell wall homeostasis in standard laboratory conditions, it remains possible that the *C. difficile* PBP1 IDRs become more important for bacterial growth or cell morphology in non-standard laboratory conditions, such as upon cell envelope stress. Indeed, in *B. subtilis*, the *pbp1* IDR mutant exhibits a more severe phenotype in a sensitized mutant background lacking GpsB and the other three *B. subtilis* aPBPs [47]. It is also possible that the residual WT PBP1 protein present during *pbp1*-KD seen in western blot analyses could obscure the functional requirement of PBP1’s IDRs in *C. difficile* (Fig 2D, S1A Fig), so analyzing IDR truncation mutations constructed in the native *pbp1* locus in various stress-inducing conditions could provide additional insight into these questions.

Notably, the presence of residual PBP1 in the *pbp1*-KD (Fig 2D) could explain why some *pbp1*-KD cells produce multiple septa (Fig 3B) [18,59]. *Pbp1*-KD cells may harbor incomplete septa, which are being synthesized simultaneously at a slower rate due to the low level of residual PBP1 that remains in the cell. Alternatively, the cell separation machinery may be delayed in these cells, perhaps indicating coordination between PBP1 and the cell separation enzymes. Additional tools will likely be necessary to achieve a more complete depletion of PBP1 beyond what is capable with the CRISPRi system in order to address this question. Regardless, our current system has enabled us to identify critical domains and residues in this enzyme through structure-activity relationship studies.

In contrast with the dispensability of the IDRs for PBP1 function, we determined that the GI domain is critical for PBP1 function in *C. difficile* (Fig 5). While we found that this novel structural feature is conserved in only a subset of bacterial families (Fig 1), the predicted location of the GI domain within *C. difficile* PBP1 is reminiscent of the UB2H domain in the division-associated aPBP in *E. coli*, PBP1b, which serves as a critical regulatory domain for interactions with essential PBP1b regulators such as LpoB and CpoB [39–42,79]. While the precise function of the GI domain remains unknown, we showed that PBP1^ΔGI^ is stably produced and has a similar localization profile as WT PBP1 (Fig 5). It is possible that the GI domain either allosterically activates the GTase or TPase activity of PBP1 directly or serves as an interaction site for as-yet unidentified PBP1 regulators. The conservation of the GI domain in aPBPs from multiple bacterial families indicates that the GI domain may govern aPBP function in diverse bacteria found in anaerobic environments ranging from the human body to deep-sea hydrothermal vents [53,54].

We further demonstrated that PBP1’s GTase activity is essential for PBP1 function during cell growth and division in *C. difficile* (Fig 6), consistent with prior analyses using the aPBP GTase inhibitor Moenomycin A [18]. In contrast, we found that PBP1’s TPase activity is essential for PBP1 to support cell growth but dispensable for its function during cell division (Fig 6). Notably, while a prior chemical screen using β-lactam antibiotics predicted that PBP1’s TPase activity would be dispensable for *C. difficile* growth because β-lactams that potently inhibit PBP1 TPase activity *in vitro* do not inhibit *C. difficile* growth [67], our genetic analyses establish that PBP1 TPase activity is essential for growth. We nevertheless observed that PBP1 TPase*-producing cells exhibit morphological defects and dysregulated septal and sidewall peptidoglycan synthesis (Fig 6). Intriguingly, we found that the uncontrolled septum synthesis observed in PBP1 TPase-deficient cells depends on the presence, but not the activity, of the non-essential enzyme PBP3 (Fig 7). These findings contrast with prior work showing that functionally redundant TPases supply the TPase activity needed for essential SEDS-bPBP complexes to mediate cell growth in other Gram-positive bacteria [3,68].

They also lead to the question of which enzyme(s) supply the TPase activity that mediates septum synthesis and promotes dysregulated RADA incorporation in PBP1^TPase*^/PBP3^TPase*^ cells (Fig 7). One possibility is that since the CRISPRi-KD of *pbp1* is not complete, the residual WT PBP1 present in these cells may contribute to the RADA incorporation at these sites. The elongation-specific bPBP, PBP2, may also contribute to dysregulated septal PG synthesis, but this seems less likely given the clear separation in cell elongation and division roles in *C. difficile* and other organisms [18,80–83]. Alternatively, the TPase activity supplied at these sites of dysregulated peptidoglycan synthesis may be provided by another class of enzyme: the LDTs, since *C. difficile* encodes five LDTs and requires at least one for viability [70,71,84,85]. The LDTs could supply the TPase activity at sites of aberrant septum synthesis observed in PBP1^TPase*^/PBP3^TPase*^ cells, perhaps by interacting with PBP1 or PBP3 or being recruited to these sites through another mechanism. Indeed LDT1, LDT4, and LDT5 have all been shown to localize to the septa in dividing cells, and cells depleted of all five LDTs exhibit an elongated phenotype, suggesting that one or more LDT plays a role during normal division as well [65,71].

**Fig 9:**
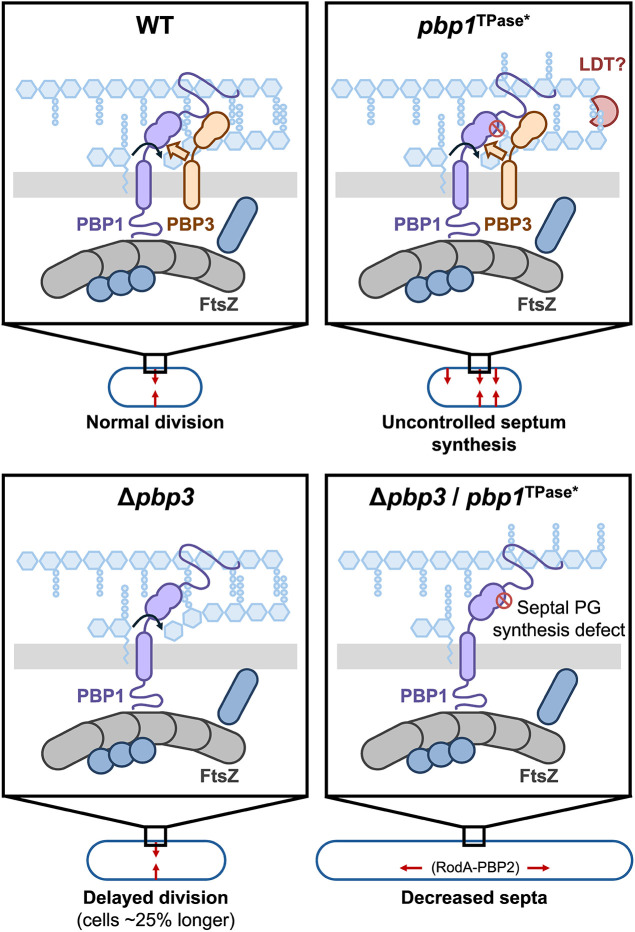
PBP3 promotes dysregulated septum synthesis by PBP1^TPase*^. PBP1 and PBP3 appear to localize to the site of division and promote septum synthesis. PBP1 is the primary driver of septal peptidoglycan synthesis in *C. difficile*. PBP3 appears to promote the ability of PBP1 to catalyze septum peptidoglycan synthesis in a non-catalytic manner. While loss of PBP3 still permits cell division, ∆*pbp3* cells are ~ 25% longer, indicating a delay in cell division. In addition, cells conditionally producing PBP1^TPase*^ exhibit uncontrolled septum peptidoglycan synthesis, which is dependent on the presence, but not the activity, of PBP3. Loss of PBP3 in cells conditionally producing PBP1^TPase*^ prevents septal peptidoglycan synthesis, leading to filamentation.

Regardless, our analyses provide novel insight into the role of PBP3 during vegetative growth in *C. difficile*. While we previously showed that PBP3 is a non-essential, sporulation-induced bPBP that promotes asymmetric division [73], the current study reveals that PBP3 functions to promote septum synthesis during vegetative growth because PBP1 TPase-deficient cells filament in the absence of PBP3 (Figs 7 and 9). While this cryptic function was uncovered in a PBP1 TPase-deficient strain, it is consistent with our prior finding that Δ*pbp3* cells are slightly longer than WT *C. difficile* during vegetative growth [18] (Fig 9). Thus, PBP3 appears to specifically modulate septal peptidoglycan synthesis by PBP1 during both asymmetric and vegetative cell division.

Consistent with this interpretation, we previously showed that PBP3 interacts directly with PBP1 when expressed in *E. coli* in a bacterial two-hybrid assay and that PBP1 co-immunoprecipitates with PBP3 during sporulation [73]. In this study, we found that PBP1 also co-immunoprecipitates with PBP3 during vegetative growth (Fig 8C) and localizes to vegetative septa (Figs 8 and 9). Similar interactions between bPBPs and aPBPs have been reported in γ-Proteobacteria to promote divisome and elongasome function [45,86,87]. For instance, the *Acinetobacter baumanii* aPBP PBP1A is part of a complex containing the division-associated bPBP, FtsI, and this complex promotes septum synthesis [87]. However, it should be noted that *A. baumanii* differs from *C. difficile* because its aPBP activity alone cannot drive septum synthesis [87]. Additionally, the division-associated *E. coli* aPBP, PBP1b, interacts directly with FtsI, leading to its recruitment to the mid-cell during division [45], while the elongasome-associated bPBP in *E. coli*, PBP2, directly stimulates PBP1a GTase activity, and the combination of these bPBP and aPBP enzymes cooperatively enhance their TPase activity [86]. Therefore, bPBPs can directly stimulate aPBP enzymatic activity. Given these observations, *C. difficile* PBP3 could directly stimulate the enzymatic activity of PBP1 such that PBP3 induces uncontrolled GTase activity in PBP1^TPase*^ cells, leading to dysregulated peptidoglycan synthesis and aberrant septa formation (Fig 9). Future investigation into the cross-talk between PBP1 and PBP3 in *C. difficile* will generate new insight into the interplay between PBP enzymes in bacteria.

## Materials and methods

### Bacterial strains and growth conditions

All *C. difficile* strains are derived from the 630Δ*erm* strain background and listed in S1 Table. Chromosomally-encoded mutations were generated in a Δ*pyrE* strain using *pyrE*-based allele coupled exchange as previously described [60]. *C. difficile* was cultured in brain heart infusion medium supplemented with 0.5% yeast extract and 0.1% L-cysteine (BHIS) with thiamphenicol (5 μg/mL) to maintain episomal plasmids, and/or kanamycin (50 μg/mL) and cefoxitin (8 μg/mL) as needed for genetic manipulation. *C. difficile* defined medium (CDDM) [88] was used for allele-coupled exchange to select for *pyrE* restoration. *C. difficile* cultures were grown at 37°C in an anaerobic chamber using a gas mixture containing 85% N_2_, 5% CO_2_, and 10% H_2_.

Plasmids used in this study were cloned using Gibson assembly, maintained in *E. coli* DH5α, and confirmed by Sanger or nanopore sequencing. *E. coli* strains containing plasmids used in this study are listed in S2 Table, with links to plasmid maps containing primer sequences used for cloning. To introduce constructs into *C. difficile*, plasmids were first transformed into *E. coli* HB101/pRK24 and then conjugated into *C. difficile* as previously described [89]. *E. coli* cultures were grown in LB at 37°C supplemented as needed with chloramphenicol (20 μg/mL), ampicillin (100 μg/mL), or kanamycin (30 μg/mL).

### Conditional expression of *pbp1* and growth curve analysis

*C. difficile* starter cultures were grown for ~2 hr, back-diluted 1:20 into BHIS with 0% or 2.5% xylose, and grown for an additional 2 hr to pre-deplete PBP1 using the xylose-inducible CRISPRi system. Cultures were then adjusted to an OD_600_ of 0.05 in media containing 0% or 2.5% xylose and/or 5 ng/mL aTc to induce the *pbp1* complementation constructs, immediately added to a 96-well plate, and the OD_600_ was measured every 30 minutes in an Epoch 2 plate reader (BioTek) in an anaerobic chamber. The xylose and aTc concentrations were titrated to identify the optimal concentrations of xylose to efficiently knock-down endogenous *pbp1* expression and aTc to allow for trans-complementation. For fluorescence microscopy and western blot analysis, the cultures were allowed to grow an additional 4 hr before samples were taken. The concentration of vehicle (water for xylose and ethanol for aTc) were normalized across all samples.

### Fluorescent probes

The fluorescent D-amino acids RADA or HADA (Tocris) were used to label *de novo* peptidoglycan synthesis and/or remodeling. Hoechst 33342 (Molecular Probes) was used to stain bacterial DNA. 500 μL of *C. difficile* culture were exposed to 50 μM RADA/HADA and/or 20 μg/mL Hoechst for 10 min, then fixed with a mixture of 100 μL 16% paraformaldehyde and 20 μL 1 M NaPO_4_ buffer (pH 7.4) for 30 min at room temperature and 30 min on ice [90]. Fixed cells were then washed three times with 1 mL 1XPBS before imaging.

### Protein localization with mScarlet-I3 fusions

*C. difficile* strains were engineered to express chromosomally-encoded P_*tet*_-*mScarlet-I3-pbp1*, P_*tet*_-*mScarlet-I3-pbp3*, or P_*tet*_-*ftsZ*-*mScarlet-I3* constructs downstream of *pyrE*. Strains were cultured to logarithmic phase in BHIS, then exposed to 5 ng/mL aTc (for *pbp1* and *pbp3* constructs) or 0.5 ng/mL aTc (for *ftsZ* construct) for 1 hr. Induced cultures were labeled with HADA and fixed as described above. After cell fixation, cells were incubated overnight at room temperature in the dark to allow chromophore maturation prior to imaging, as previously described [90].

### Fluorescence microscopy

Microscopy samples were imaged on agarose pads (1% agarose in 1XPBS). Phase-contrast and fluorescence micrographs were acquired with a Leica DMi8 inverted microscope equipped with a 63X 1.4 NA Plan Apochromat oil-immersion phase-contrast objective, a high precision motorized stage (Pecon), and in a 37°C incubator (Pecon). Excitation light was generated by a Lumencor Spectra-X multi-LED light source with integrated excitation filters. An XLED-QP quadruple-band dichroic beam-splitter (Leica) was used (transmission: 415, 470, 570, and 660 nm) with an external filter wheel for all fluorescent channels. HADA and Hoechst were excited at 395/25, and emitted light was filtered using a 440/40-nm emission filter, with 120 ms (HADA) or 100 ms (Hoechst) exposure times; RADA and mScarlet-I3 were excited at 550/28 nm, and emitted light was filtered using a 590/50-nm emission filter, with 300 ms (RADA) or 150 ms (mScarlet-I3) exposure times. Light was detected using a Leica DFC 9000 GTC sCMOS camera. 1–2 μm z-stacks were taken with 0.21 μm z-slices. Images were acquired using the LASX software, and fluorescence images were deconvolved using Leica Small Volume Computational Clearing with the following settings: refractive index 1.33, strength 60%, and regularization 0.05.

Images were processed using FIJI to select the best-focused z-plane for each channel and adjust the image brightness and contrast of images. Identical minimum and maximum values were applied to a given channel across all images in a figure panel so that direct comparisons can be made across images within a figure panel, unless otherwise specified in the figure legend. Demographs were generated with MicrobeJ in FIJI, using segmentation masks generated by SuperSegger [91].

### Quantification of cell length and width

To quantify the length and width of cells, phase-contrast images were segmented using the MATLAB-based image analysis pipeline SuperSegger [91]. To enable analysis of filamentous cells, the default “60xec” settings were modified with the following: CONST.superSeggerOpti.MAX_WIDTH = 1e10; and CONST.seg.OPTI_FLAG = false. Cells cut off by the edge of the image were excluded from the analysis and the minimum cell length was set to 2 μm to avoid inadvertently including cellular debris in the dataset.

### Western blot analysis

*C. difficile* cultures were pelleted, resuspended in 25 μL 1XPBS, freeze-thawed three times, mixed with 25 μL EBB buffer (9 M urea, 2 M thiourea, 4% SDS, 2 mM β-mercaptoethanol), and boiled for 20 min to lyse cells. To normalize the protein load across samples, the lysates were diluted based on the starting OD_600_ of the sample prior to lysis, and then the same volume of lysate was run using SDS-polyacrylamide gel electrophoresis (SDS-PAGE) on a 10% polyacrylamide gel. Proteins were transferred to polyvinylidene difluoride membranes, which were subsequently probed with primary antibodies: rabbit polyclonal anti-PBP1 TF134 [73] at 1:1,000 dilution; rabbit polyclonal anti-DivIVA TF142 (this study) at 1:1,000 dilution; chicken polyclonal anti-GDH (aCdGDH; Thermo) at 1:10,000 dilution; and/or a mouse monoclonal M2 anti-FLAG antibody (Sigma) at 1:5,000. Anti-rabbit, anti-chicken, and anti-mouse IR800 or IR680 secondary antibodies (LI-COR Biosciences, 1:30,000) were used to detect bands with a LI-COR Odyssey CLx imaging system. Quantification of westerns was performed using LI-COR imaging software Image Studio Lite with background subtraction. Band intensities were normalized to GDH for each sample, and further normalized to the vehicle-treated control to calculate the fold-change in PBP1 levels relative to the control cells.

### Antibody production

The anti-DivIVA antibody used for western blots in this study were raised in rabbits by Cocalico Biologicals against *C. difficile* DivIVA-His_6_ purified from *E. coli*. DivIVA-His_6_ was produced in BL21(DE3) *E. coli* harboring pET28a-*divIVA*-*His*_*6*_ and purified by Ni^2+^-affinity purification as previously described [92]. Proteins were purified further by size exclusion chromatography (SEC) in a buffer containing 200 mM NaCl, 10 mM Tris-HCl pH 7.5, 5% glycerol, and 1 mM DTT, using a Superdex 200 Increase 10/300 GL (GE Healthcare) column. SEC fractions containing DivIVA, as determined by SDS-PAGE and western blotting with an anti-His_6_ antibody (HIS.H8; Thermo), were combined, concentrated, and used to raise antibodies. Antisera reactivity and specificity for DivIVA was validated by western blot against *C. difficile* lysate from WT and a *divIVA* CRISPRi knock-down strain.

### Co-immunoprecipitation (Co-IP) analysis

To immunoprecipitate FLAG-tagged proteins from vegetatively growing *C. difficile*, 200 μL of exponentially growing cultures of *C. difficile* harboring a xylose-inducible P_*xyl*_-*pbp3*-FLAG_3_ or a P_*xyl*_-*pbp3* un-tagged expression construct engineered downstream of the *pyrE* locus in the chromosome were spread on BHIS agar media containing 0.5% xylose and grown for 9 hr. Cells were scraped from 3 plates per strain and pooled in 750 μL of BHIS. A portion of cells was visualized by microscopy with RADA labeling to confirm that cells were actively dividing and forming septa. Co-immunoprecipitation was performed similar to previously described methods. Crosslinking of cells was performed with 0.25% final concentration of PFA for 15 minutes at 37°C followed by quenching with 350 mM glycine for 10 minutes on ice. Cells were then pelleted, re-suspended in 750 µL of FLAG IP Buffer (150 mM NaCl, 50 mM Tris-HCl pH 7.5), transferred to screwcap tubes containing MP Biomedicals Lysing Matrix E, and frozen at -80°C. Cell pellets were thawed and bead beat on an MP Biomedicals FastPrep-24 four times at 5.5 M/second for 1 minute for a total of four rounds of lysis, with 5 minutes on ice between rounds of bead-beating. Next, 1X HALT protease inhibitors and 0.5% final concentration of dodecyl-β-d-maltoside (DDM) detergent was added to lysed cells, and cell lysates were rotated at room temperature for 1 hour to solubilize membrane proteins. Lysates were clarified with centrifugation at 10,000 × g for 1 minute, and 200 µL of pre-equilibrated Anti-FLAG M2 magnetic resin was added to the clarified lysate. The slurry containing lysate and resin was rotated for 1 hour at room temperature. To remove unbound proteins, the resin was washed three times briefly and once for 15 minutes with 1 mL FLAG IP Buffer containing 0.5% DDM. This step was repeated a total of two times. Then, the resin was washed three times briefly and once for 15 minutes with FLAG IP Buffer containing no detergent. Finally, bound proteins were eluted from the Anti-FLAG resin with 100 μg/mL 3XFLAG peptide. Samples were boiled in 1X sample buffer (40% glycerol, 1M Tris pH 6.8, 20% β-mercaptoethanol, 8% SDS, and 0.04% bromophenol blue) for 10 minutes to reverse crosslinks and then analyzed by western blot.

## Supporting information

S1 Fig Analysis of PBP1 levels in and the cell morphology of *pbp1* trans-complementation strains. **(A)** Quantification of western blots derived from *C. difficile* strains containing the xylose-inducible *pbp1*-KD construct and plasmid-encoded aTc-inducible *gusA*, *pbp1*_WT_, or *pbp1*_im_ complementation constructs cultured in the presence and absence of 2.5% xylose and/or 5 ng/mL aTc as indicated in the scheme in Fig 2C. PBP1 levels were normalized to GDH for each sample, and the fold-change in PBP1 was calculated relative to the vehicle-treated control. Mean and standard error were calculated across three independent experiments. Representative western blots are found in Fig 2D. (**B**) Quantification of cell width for >1200 cells across three independent experiments. Dots indicate individual cells, and the larger, outlined symbols represent the mean cell length from each replicate. The mean and standard deviation were calculated across replicates; statistical significance was determined by a one-way ANOVA with Tukey’s multiple comparisons test. ns, not significant; *p < 0.05; **p < 0.01; ***p < 0.001; ****p < 0.0001.
(TIF)

S2 Fig PBP1 depletion results in a population of cells with decreased RADA fluorescence. **(A)** *C. difficile* harboring the *pbp1*-KD cassette and pRPF185-P_tet_-*gusA* was cultured in the presence and absence of xylose and/or aTc per the scheme indicated in Fig 2C, and cells were labeled with RADA for 10 min, fixed, and imaged by fluorescence microscopy. The mean RADA fluorescence was quantified for each cell using SuperSegger. A histogram depicting the percent of cells with the indicated amount of RADA fluorescence reveals that PBP1 depletion results in a population with decreased RADA signal. (**B**) The indicated strains were cultured in the presence of both xylose and aTc, and RADA fluorescence was quantified as in panel A. Note that the P_tet_-*gusA* data from panel A (blue squares) was duplicated in panel B for comparison. >1300 cells were quantified across three independent experiments, and symbols represent the mean and standard error of the mean from three replicates.
(TIF)

S3 Fig PBP1 over-production increases C. difficile cell width. **(A–E)** Logarithmically growing C. difficile harboring the *pbp1*-KD cassette and pRPF185-P_tet_-*pbp1*_im_ was cultured in the presence of the indicated concentration of aTc for 4 hours. Cells were then fixed and imaged. (**A**) Phase-contrast images representative of three independent experiments are shown. The yellow arrows highlight cell lysis, which was frequently observed at the highest concentration of aTc (50 ng/mL). Scale bar = 5 μm. (**B**) Cell length and (**C**) cell width were quantified for >2500 cells using SuperSegger. The small grey dots represent individual cells, and the larger symbols represent the mean from three independent experiments color coded according to the experiment. (D) Western blot quantification for PBP1 normalized to GDH is shown with the fold-change calculated relative to the 0 ng/mL aTc control. (E) The cell width (data from panel C) plotted against the PBP1 protein fold-change (data from panel D) reveals that cell width correlates with PBP1 levels for 0-25 ng/mL aTc. The 50 ng/mL aTc sample exhibited substantial cell lysis. (F) The baseline PBP1 levels in C. difficile harboring the *pbp1*-KD cassette with pRPF185-P_tet_-*gusA* or pRPF185-P_tet_-*pbp1*_im_ were calculated relative to WT *C. difficile*. There is no significant difference in the baseline PBP1 levels in the mutant strains without aTc addition. For panels B–D, a one-way ANOVA with Dunnett’s post-test was performed to test for statistically significant differences. ns, not significant; *p < 0.05; **p < 0.01; ***p < 0.001; ****p < 0.0001.
(TIF)

S4 Fig Impact of N- and C-terminal IDR mutations on PBP1 levels and cell morphology. (A) Quantification of western blots derived from *C. difficile* strains containing the xylose-inducible *pbp1*-KD construct and plasmid-encoded aTc-inducible *pbp1*_im_^ΔNT^, *pbp1*_im_^ΔCT^, or *pbp1*_im_^ΔNTCT^ complementation constructs cultured in the presence and absence of 2.5% xylose and/or 5 ng/mL aTc as indicated in the scheme in Fig 2C. PBP1 levels were normalized to GDH for each sample, and the fold-change in PBP1 was calculated relative to the vehicle-treated control. Mean and standard error were calculated across three independent experiments. Representative western blots are found in Fig 4B. (B) Quantification of the length of >2600 cells across three independent experiments using SuperSegger [91]. Dots indicate cells, and the larger, outlined symbols represent the mean cell length from each replicate. The mean and standard deviation were calculated across replicates; statistical significance was determined by a one-way ANOVA with Tukey’s multiple comparisons test. ns, not significant; *p < 0.05; **p < 0.01; ***p < 0.001; ****p < 0.0001.
(TIF)

S5 Fig Curvature of PBP1 trans-complementation strains lacking N- and or C-terminal IDRs. (A) C. difficile containing the xylose-inducible *pbp1*-KD construct and aTc-inducible *pbp1*_im_, *pbp1*_im_^ΔNT^, *pbp1*_im_^ΔCT^, or *pbp1*_im_^ΔNTCT^ complementation constructs were cultured in the presence of both 2.5% xylose and 5 ng/mL aTc as indicated in the scheme in Fig 2C to conditionally express the indicated pbp1 construct. The curvature of >4500 cells across four independent replicates was analyzed by MicrobeJ. Each cell is indicated by a dot, and the larger, outlined symbols represent the mean across replicates. The dotted line indicates the mean of the WT control (*pbp1*_im_), highlighting that cells conditionally expressing *pbp1*_im_^ΔCT^ or *pbp1*_im_^ΔNTCT^ exhibit a modest increase in curvature above the control. The mean and standard deviation were calculated across replicates, and a one-way ANOVA with Tukey’s post-test was used to test statistical significance between groups; ns, not significant. (B) A histogram of the data in panel A is represented, indicating the percentage of cells that exhibit the indicated level of curvature, n = 4. Data was log-transformed before performing a two-way ANOVA with Tukey’s post-test was used to test statistical significance between the curves: ns, not significant; *, *p* < 0.05.
(TIF)

S6 Fig Alphafold3 structural prediction of the WT (A) or ΔGI (B) PBP1 protein. Indicated in pink is the 66-amino acid GTase-associated insertion (GI) domain, as identified in the protein sequence alignment in Fig 2C. To construct the ΔGI mutant, we performed Alphafold3 modeling with various deletions to identify a variant that lacked the GI domain with a minimal impact on the overall architecture of the protein. Based on this analysis, residues 267-328 were deleted in the ΔGI mutant, leaving behind four residues from the originally identified domain outlined in panel B.
(TIF)

S7 Fig Impact of a ΔGI mutation on PBP1 levels. Quantification of western blots derived from *C. difficile* strains containing the xylose-inducible *pbp1*-KD construct and a plasmid-encoded aTc-inducible *pbp1*_im_^ΔGI^ complementation construct cultured in the presence and absence of 2.5% xylose and/or 5 ng/mL aTc as indicated in the scheme in Fig 2C. PBP1 levels were normalized to GDH for each sample, and the fold-change in PBP1 was calculated relative to the vehicle treated control. Mean and standard error were calculated across three independent experiments. Representative western blots are found in Fig 5B. The mean and standard deviation were calculated across replicates; statistical significance was determined by a one-way ANOVA with Tukey’s multiple comparisons test. ns, not significant; *p < 0.05; **p < 0.01.
(TIF)

S8 Fig Impact of GTase* and TPase* mutations on PBP1 levels. Quantification of western blots for *C. difficile* containing the xylose-inducible *pbp1*-KD construct and aTc-inducible *pbp1*_im_^GTase*^ (left) or *pbp1*_im_^TPase*^ (right) complementation constructs cultured in the presence and absence of 2.5% xylose and/or 5 ng/mL aTc as indicated in the scheme in Fig 2C. PBP1 levels were normalized to GDH for each sample, and the fold-change in PBP1 was calculated relative to the vehicle treated control. Mean and standard error were calculated across three independent experiments. Statistical significance was determined by a one-way ANOVA with Tukey’s multiple comparisons test. ns, not significant; *p < 0.05; **p < 0.01. Representative western blots are found in Fig 6B.
(TIF)

S9 Fig PBP1^TPase*^-producing *C. difficile* have puncta of aberrant RADA incorporation. *C. difficile* containing the xylose-inducible *pbp1*-KD construct and aTc-inducible *pbp1*_im_, *pbp1*_im_^GTase*^, or *pbp1*_im_^TPase*^ complementation constructs were cultured in the presence of both 2.5% xylose and 5 ng/mL aTc as indicated in the scheme in Fig 2C to conditionally express the indicated pbp1 construct. Aberrant puncta of RADA signal were scored across three independent experiments for a total of >500 bacteria. Grey dots indicate individual cells, and the larger, black symbols represent the mean cell length from each replicate. The mean and standard deviation were calculated across replicates; statistical significance was determined by a one-way ANOVA with Tukey’s multiple comparisons test. *p < 0.05; **p < 0.01; ****p < 0.0001.
(TIF)

S10 Fig RADA incorporation in C. difficile PBP1 catalytic site mutants. (**A** and **B**) *C. difficile* strains harboring the *pbp1*-KD cassette and pRPF185 plasmids encoding (A) P_tet_-pbp1_im_^GTase*^ (*n* = 3), P_tet_-*pbp1*_im_^ΔGI^ (*n* = 3), or (B) P_tet_-*pbp1*_im_^TPase*^ in either the WT (*n* = 7) or Δ*pbp3* (*n* = 4) background were cultured in the presence of both 2.5% xylose and 5 ng/mL aTc as indicated in the scheme in Fig 2C to conditionally express the indicated *pbp1* construct. The cells were then labeled with RADA for 10 min, fixed, and imaged by fluorescence microscopy. Data from P_tet_-*pbp1*_im_ (black circles) is the same data shown in Supplementary S2B Fig and reproduced here in both panels for the sake of comparison. The mean RADA fluorescence was quantified for each cell using SuperSegger. >690 cells were quantified across 3–7 independent experiments and symbols represent the mean and standard error of the mean across replicates.
(TIF)

S11 Fig Analysis of PBP1 levels in TPase-deficient pbp1 trans-complementation strains. Western blot analyses of PBP1 in WT or Δpbp3 cells harboring *pbp1*-KD and *pbp1*^TPase*^ complementation construct exposed to vehicle or 2.5% xylose and/or 5 ng/mL aTc per the scheme in Fig 2C. GDH (46.0 kDa) was used as a load control. Blots are representative of two independent experiments.
(TIF)

S1 Table *C. difficile* strains used in this study. (XLSX)

S2 Table *E. coli* strains used in this study. (XLSX)
