# Supplementary figures and images for: Molecular dissection of Class A PBP function uncovers novel features of the non-canonical Clostridioides difficile divisome complex
Source: PLoS Genet. 2025 Oct 21;21(10):e1011746. doi: 10.1371/journal.pgen.1011746 (PMC12539705; doi:10.1371/journal.pgen.1011746)

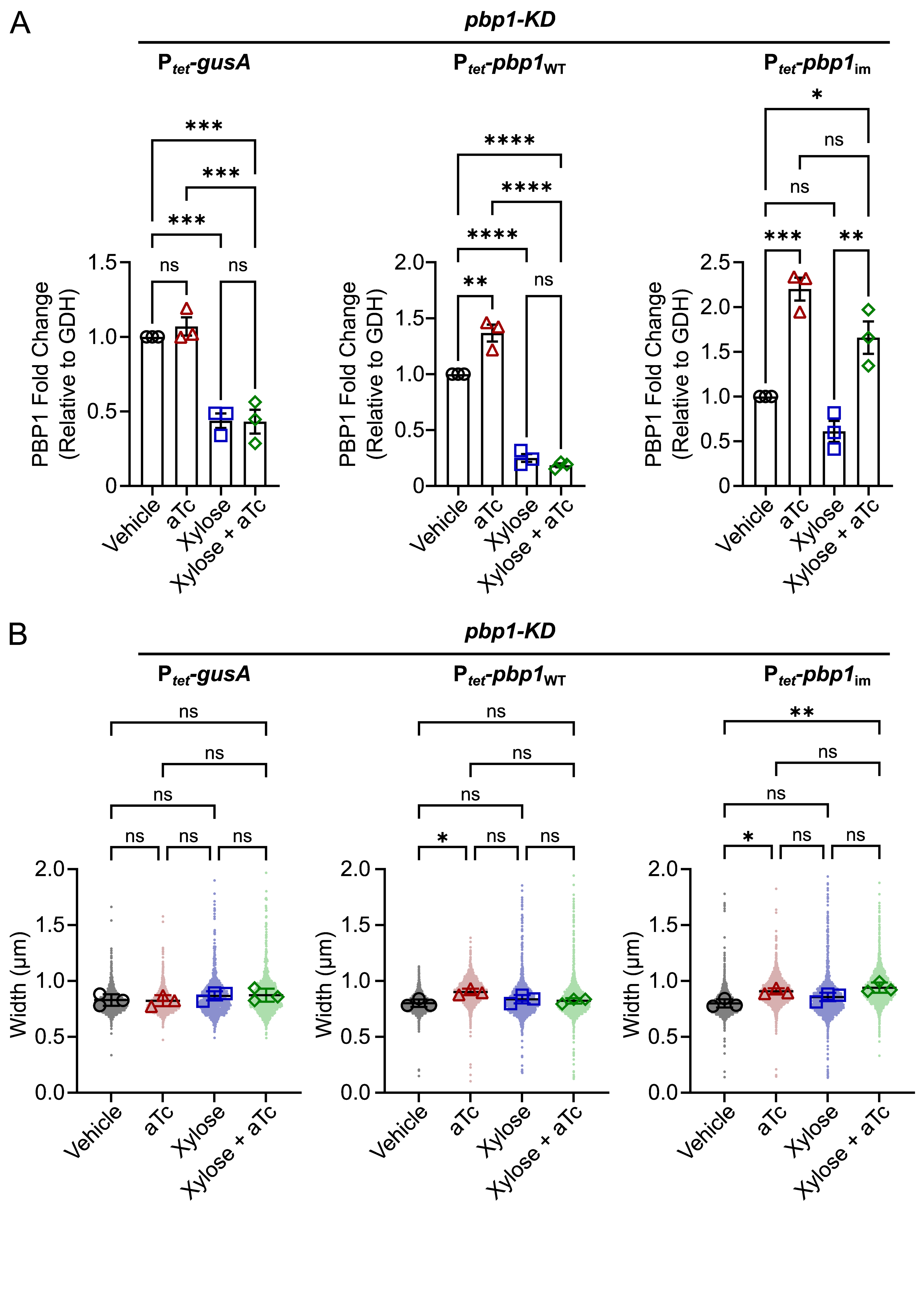

Supplement: S1 Fig — (A) Quantification of western blots derived from C. difficile strains containing the xylose-inducible pbp1-KD construct and plasmid-encoded aTc-inducible gusA, pbp1WT, or pbp1im complementation constructs cultured in the presence and absence of 2.5% xylose and/or 5 ng/mL aTc as indicated in the scheme in Fig 2C. PBP1 levels were normalized to GDH for each sample, and the fold-change in PBP1 was calculated relative to the vehicle-treated control. Mean and standard error were calculated across three independent experiments. Representative western blots are found in Fig 2D. (B) Quantification of cell width for >1200 cells across three independent experiments. Dots indicate individual cells, and the larger, outlined symbols represent the mean cell length from each replicate. The mean and standard deviation were calculated across replicates; statistical significance was determined by a one-way ANOVA with Tukey’s multiple comparisons test. ns, not significant; *p < 0.05; **p < 0.01; ***p < 0.001; ****p < 0.0001. (TIF) [file pgen.1011746.s001.tif]

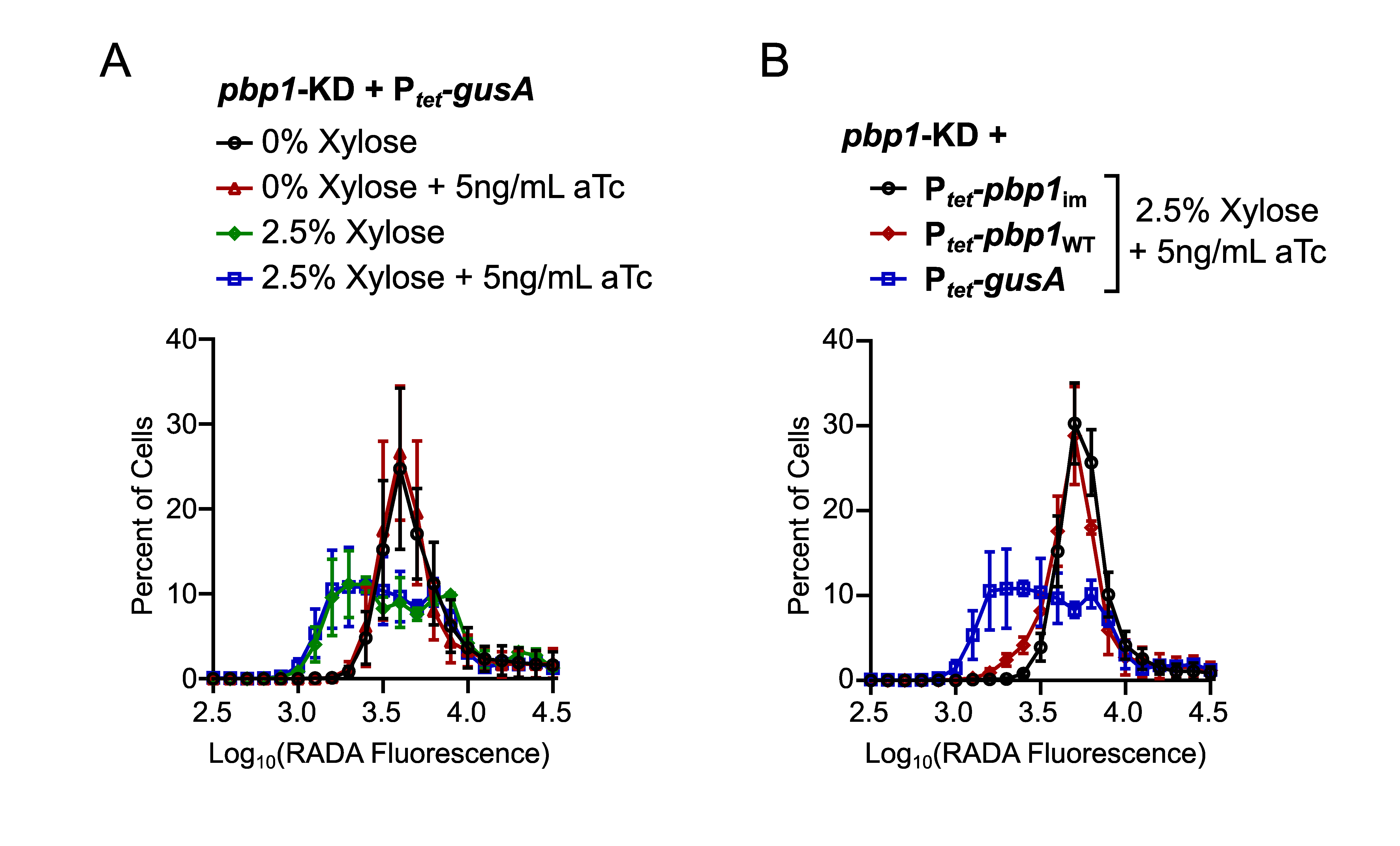

Supplement: S2 Fig — (A) C. difficile harboring the pbp1-KD cassette and pRPF185-Ptet-gusA was cultured in the presence and absence of xylose and/or aTc per the scheme indicated in Fig 2C, and cells were labeled with RADA for 10 min, fixed, and imaged by fluorescence microscopy. The mean RADA fluorescence was quantified for each cell using SuperSegger. A histogram depicting the percent of cells with the indicated amount of RADA fluorescence reveals that PBP1 depletion results in a population with decreased RADA signal. (B) The indicated strains were cultured in the presence of both xylose and aTc, and RADA fluorescence was quantified as in panel A. Note that the Ptet-gusA data from panel A (blue squares) was duplicated in panel B for comparison. >1300 cells were quantified across three independent experiments, and symbols represent the mean and standard error of the mean from three replicates. (TIF) [file pgen.1011746.s002.tif]

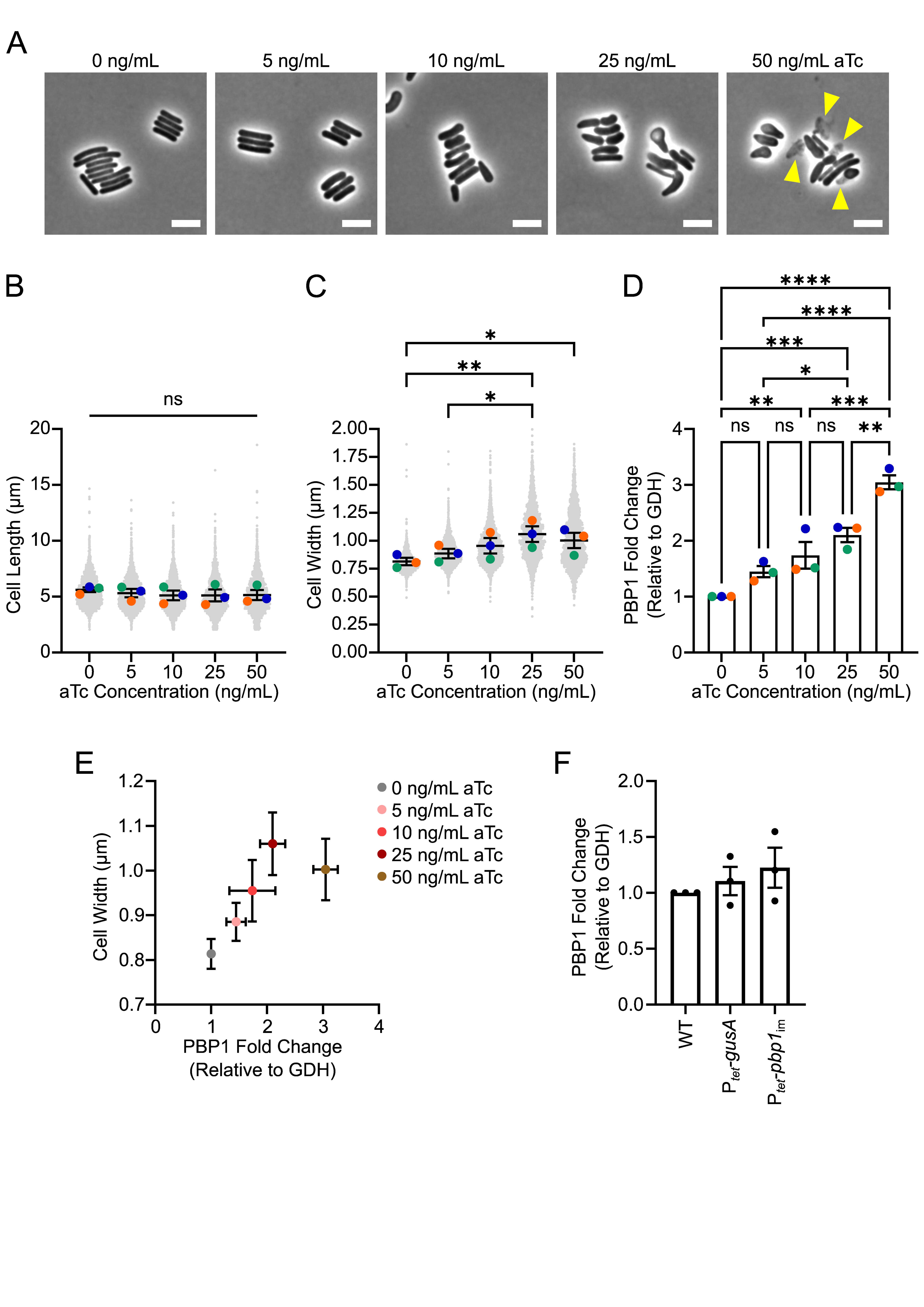

Supplement: S3 Fig — (A–E) Logarithmically growing C. difficile harboring the pbp1-KD cassette and pRPF185-Ptet-pbp1im was cultured in the presence of the indicated concentration of aTc for 4 hours. Cells were then fixed and imaged. (A) Phase-contrast images representative of three independent experiments are shown. The yellow arrows highlight cell lysis, which was frequently observed at the highest concentration of aTc (50 ng/mL). Scale bar = 5 μm. (B) Cell length and (C) cell width were quantified for >2500 cells using SuperSegger. The small grey dots represent individual cells, and the larger symbols represent the mean from three independent experiments color coded according to the experiment. (D) Western blot quantification for PBP1 normalized to GDH is shown with the fold-change calculated relative to the 0 ng/mL aTc control. (E) The cell width (data from panel C) plotted against the PBP1 protein fold-change (data from panel D) reveals that cell width correlates with PBP1 levels for 0-25 ng/mL aTc. The 50 ng/mL aTc sample exhibited substantial cell lysis. (F) The baseline PBP1 levels in C. difficile harboring the pbp1-KD cassette with pRPF185-Ptet-gusA or pRPF185-Ptet-pbp1im were calculated relative to WT C. difficile. There is no significant difference in the baseline PBP1 levels in the mutant strains without aTc addition. For panels B–D, a one-way ANOVA with Dunnett’s post-test was performed to test for statistically significant differences. ns, not significant; *p < 0.05; **p < 0.01; ***p < 0.001; ****p < 0.0001. (TIF) [file pgen.1011746.s003.tif]

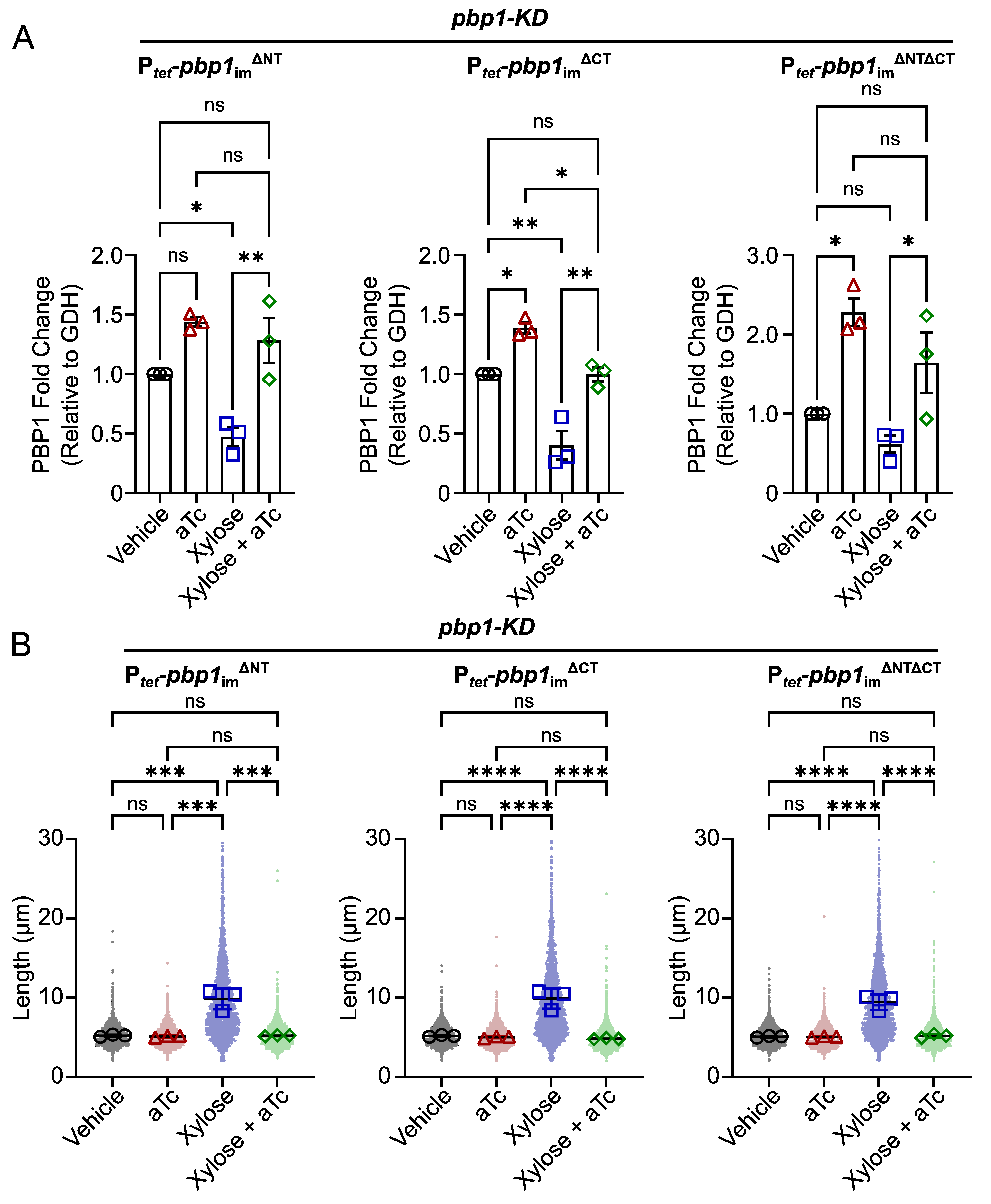

Supplement: S4 Fig — (A) Quantification of western blots derived from C. difficile strains containing the xylose-inducible pbp1-KD construct and plasmid-encoded aTc-inducible pbp1imΔNT, pbp1imΔCT, or pbp1imΔNTCT complementation constructs cultured in the presence and absence of 2.5% xylose and/or 5 ng/mL aTc as indicated in the scheme in Fig 2C. PBP1 levels were normalized to GDH for each sample, and the fold-change in PBP1 was calculated relative to the vehicle-treated control. Mean and standard error were calculated across three independent experiments. Representative western blots are found in Fig 4B. (B) Quantification of the length of >2600 cells across three independent experiments using SuperSegger [91]. Dots indicate cells, and the larger, outlined symbols represent the mean cell length from each replicate. The mean and standard deviation were calculated across replicates; statistical significance was determined by a one-way ANOVA with Tukey’s multiple comparisons test. ns, not significant; *p < 0.05; **p < 0.01; ***p < 0.001; ****p < 0.0001. (TIF) [file pgen.1011746.s004.tif]

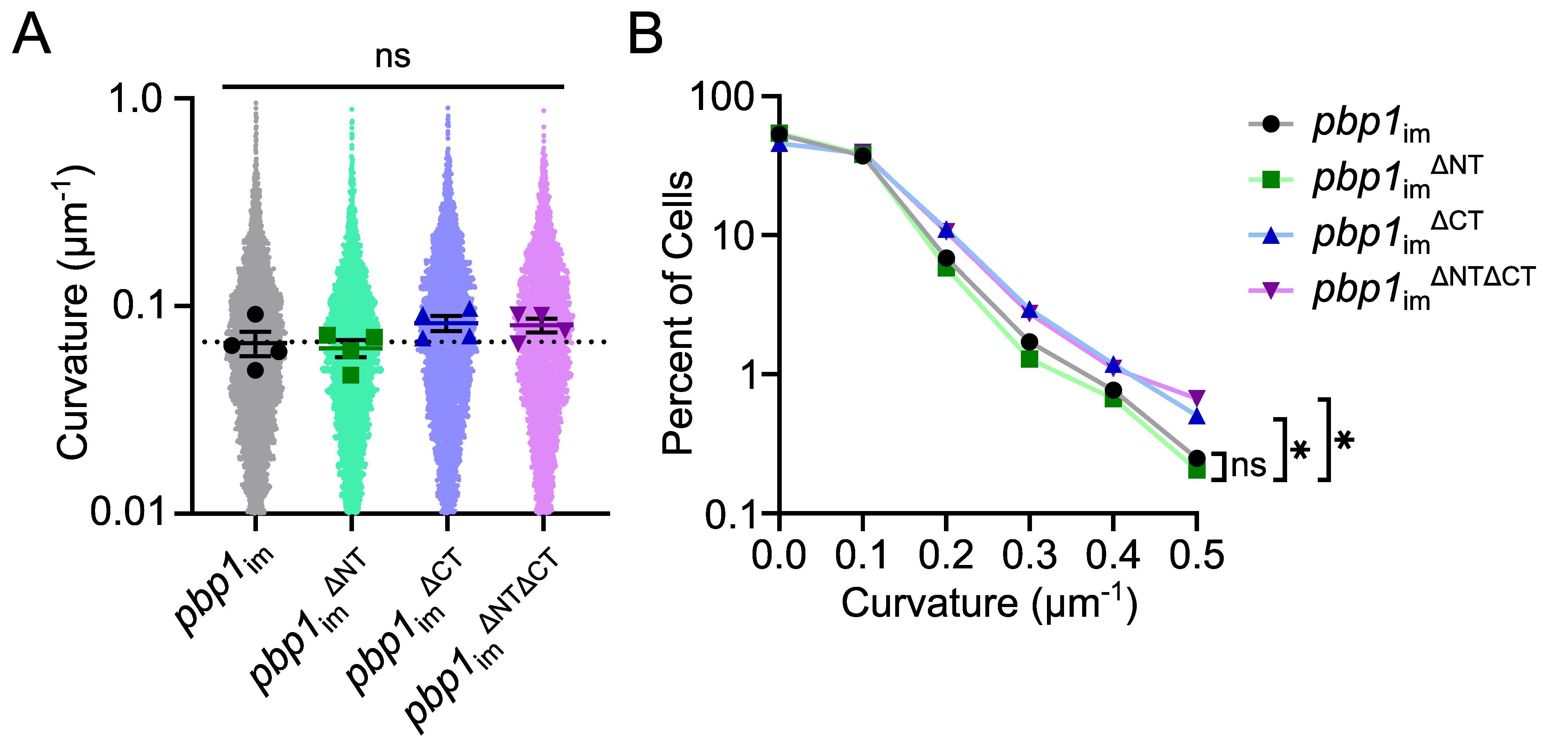

Supplement: S5 Fig — (A) C. difficile containing the xylose-inducible pbp1-KD construct and aTc-inducible pbp1im, pbp1imΔNT, pbp1imΔCT, or pbp1imΔNTCT complementation constructs were cultured in the presence of both 2.5% xylose and 5 ng/mL aTc as indicated in the scheme in Fig 2C to conditionally express the indicated pbp1 construct. The curvature of >4500 cells across four independent replicates was analyzed by MicrobeJ. Each cell is indicated by a dot, and the larger, outlined symbols represent the mean across replicates. The dotted line indicates the mean of the WT control (pbp1im), highlighting that cells conditionally expressing pbp1imΔCT or pbp1imΔNTCT exhibit a modest increase in curvature above the control. The mean and standard deviation were calculated across replicates, and a one-way ANOVA with Tukey’s post-test was used to test statistical significance between groups; ns, not significant. (B) A histogram of the data in panel A is represented, indicating the percentage of cells that exhibit the indicated level of curvature, n = 4. Data was log-transformed before performing a two-way ANOVA with Tukey’s post-test was used to test statistical significance between the curves: ns, not significant; *, p < 0.05. (TIF) [file pgen.1011746.s005.tif]

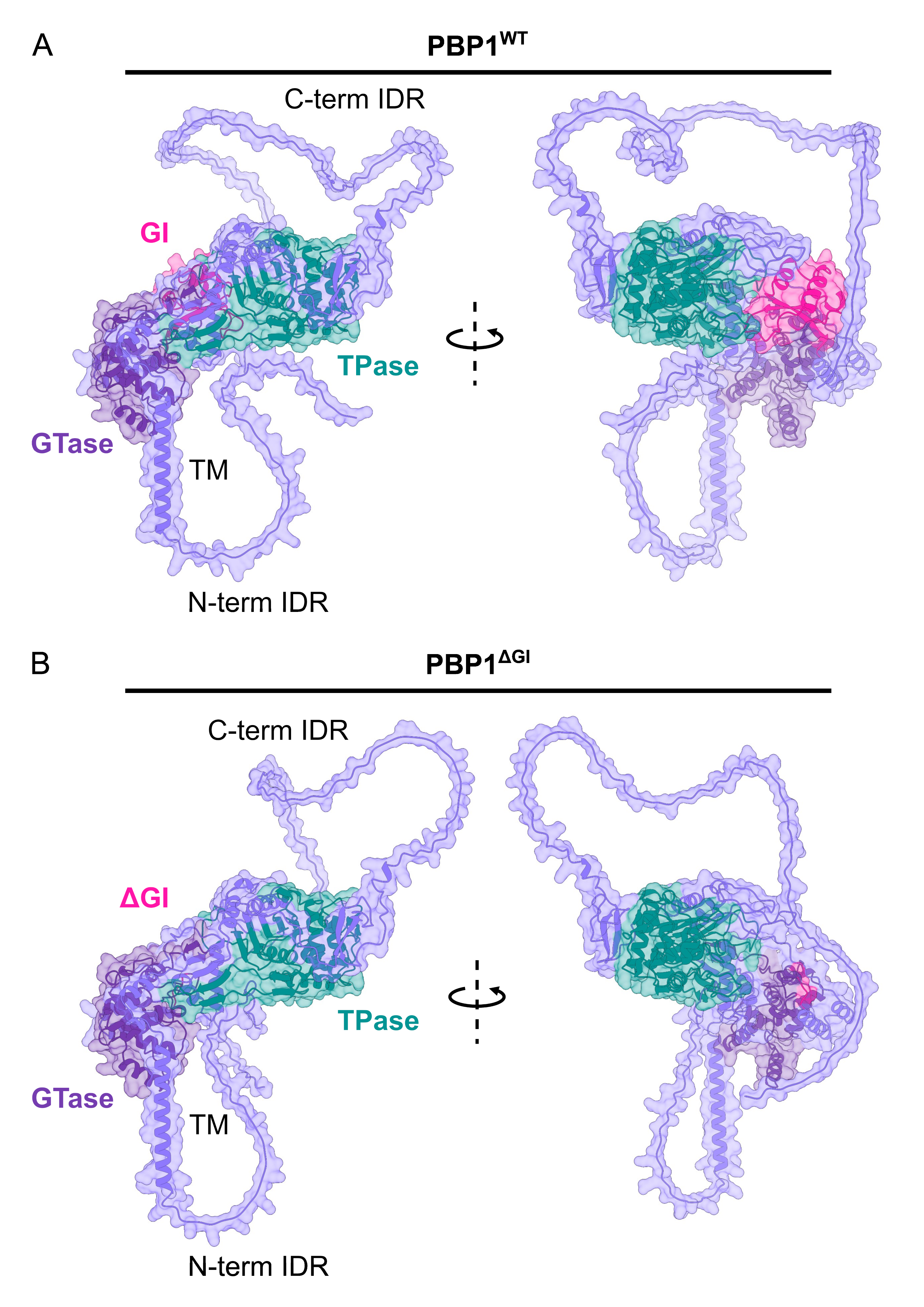

Supplement: S6 Fig — Indicated in pink is the 66-amino acid GTase-associated insertion (GI) domain, as identified in the protein sequence alignment in Fig 2C. To construct the ΔGI mutant, we performed Alphafold3 modeling with various deletions to identify a variant that lacked the GI domain with a minimal impact on the overall architecture of the protein. Based on this analysis, residues 267-328 were deleted in the ΔGI mutant, leaving behind four residues from the originally identified domain outlined in panel B. (TIF) [file pgen.1011746.s006.tif]

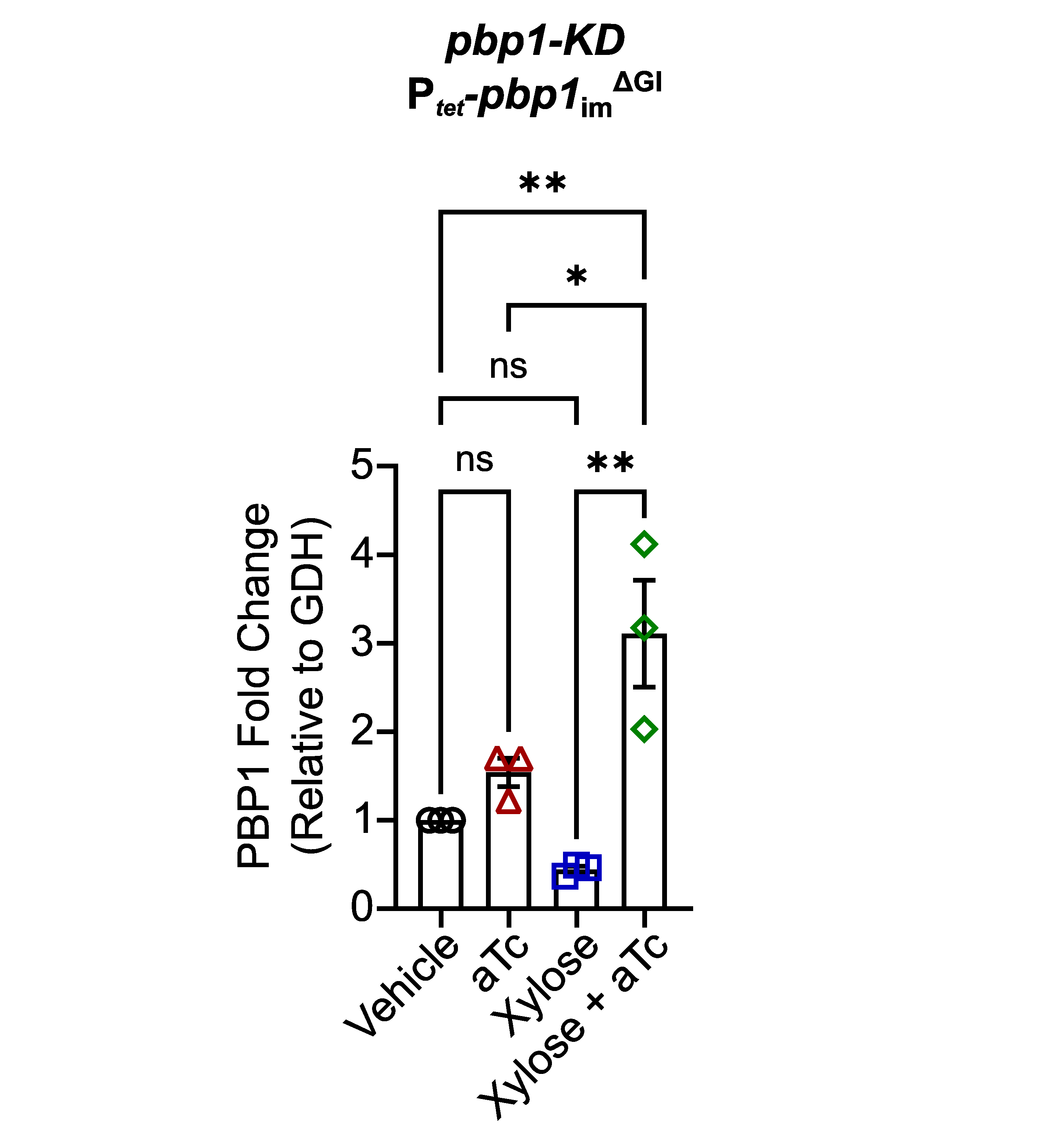

Supplement: S7 Fig — Quantification of western blots derived from C. difficile strains containing the xylose-inducible pbp1-KD construct and a plasmid-encoded aTc-inducible pbp1imΔGI complementation construct cultured in the presence and absence of 2.5% xylose and/or 5 ng/mL aTc as indicated in the scheme in Fig 2C. PBP1 levels were normalized to GDH for each sample, and the fold-change in PBP1 was calculated relative to the vehicle treated control. Mean and standard error were calculated across three independent experiments. Representative western blots are found in Fig 5B. The mean and standard deviation were calculated across replicates; statistical significance was determined by a one-way ANOVA with Tukey’s multiple comparisons test. ns, not significant; *p < 0.05; **p < 0.01. (TIF) [file pgen.1011746.s007.tif]

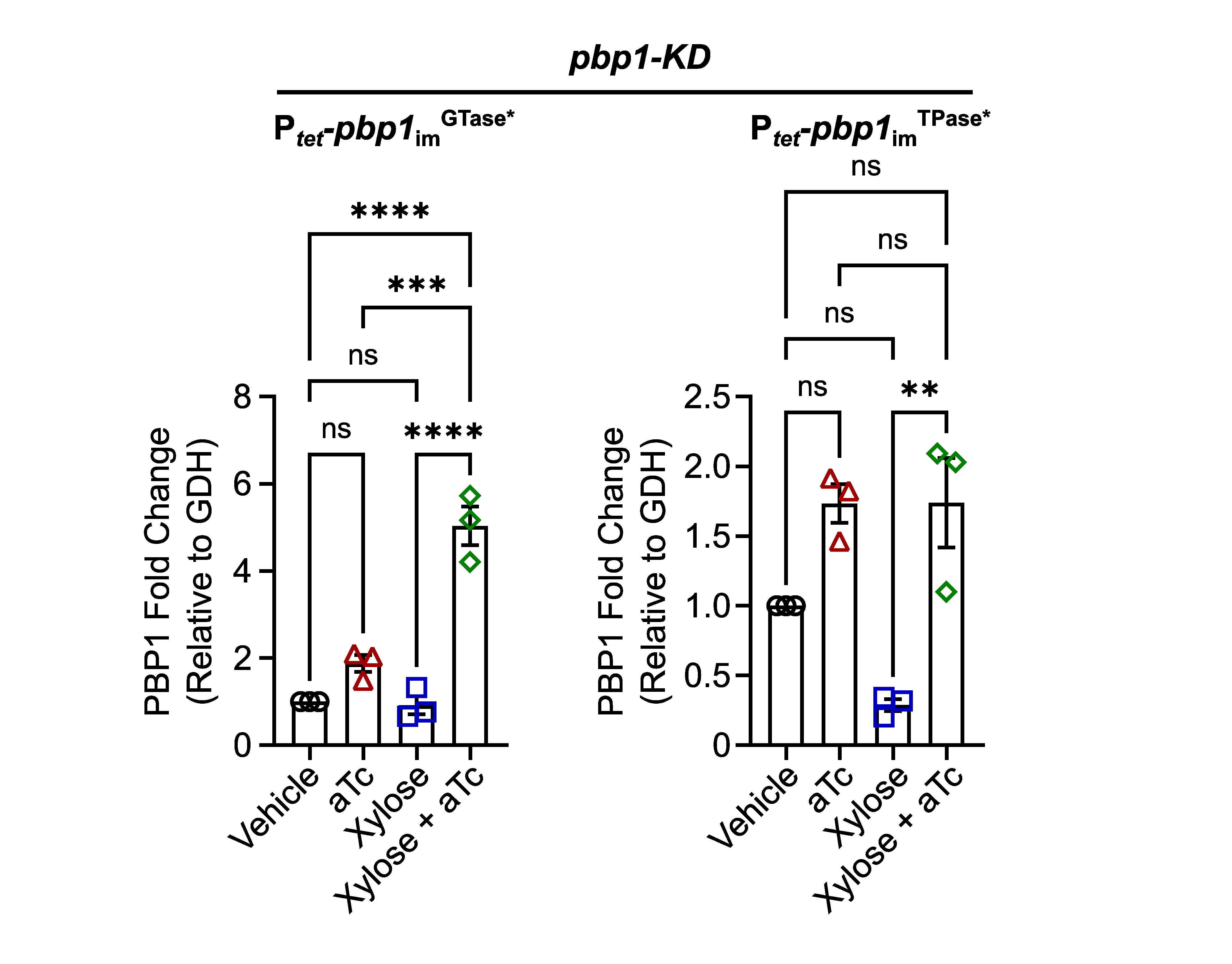

Supplement: S8 Fig — Quantification of western blots for C. difficile containing the xylose-inducible pbp1-KD construct and aTc-inducible pbp1imGTase* (left) or pbp1imTPase* (right) complementation constructs cultured in the presence and absence of 2.5% xylose and/or 5 ng/mL aTc as indicated in the scheme in Fig 2C. PBP1 levels were normalized to GDH for each sample, and the fold-change in PBP1 was calculated relative to the vehicle treated control. Mean and standard error were calculated across three independent experiments. Statistical significance was determined by a one-way ANOVA with Tukey’s multiple comparisons test. ns, not significant; *p < 0.05; **p < 0.01. Representative western blots are found in Fig 6B. (TIF) [file pgen.1011746.s008.tif]

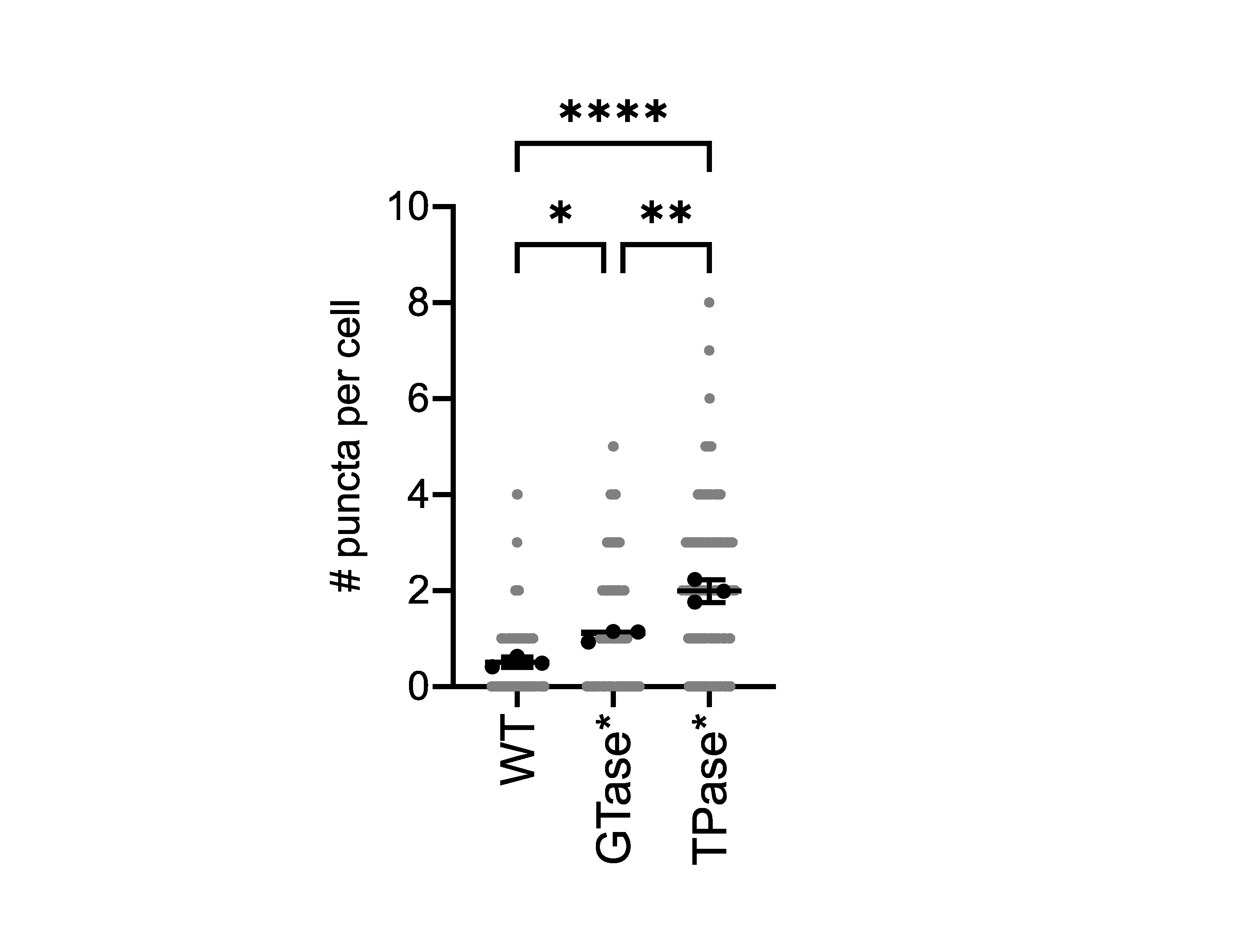

Supplement: S9 Fig — C. difficile containing the xylose-inducible pbp1-KD construct and aTc-inducible pbp1im, pbp1imGTase*, or pbp1imTPase* complementation constructs were cultured in the presence of both 2.5% xylose and 5 ng/mL aTc as indicated in the scheme in Fig 2C to conditionally express the indicated pbp1 construct. Aberrant puncta of RADA signal were scored across three independent experiments for a total of >500 bacteria. Grey dots indicate individual cells, and the larger, black symbols represent the mean cell length from each replicate. The mean and standard deviation were calculated across replicates; statistical significance was determined by a one-way ANOVA with Tukey’s multiple comparisons test. *p < 0.05; **p < 0.01; ****p < 0.0001. (TIF) [file pgen.1011746.s009.tif]

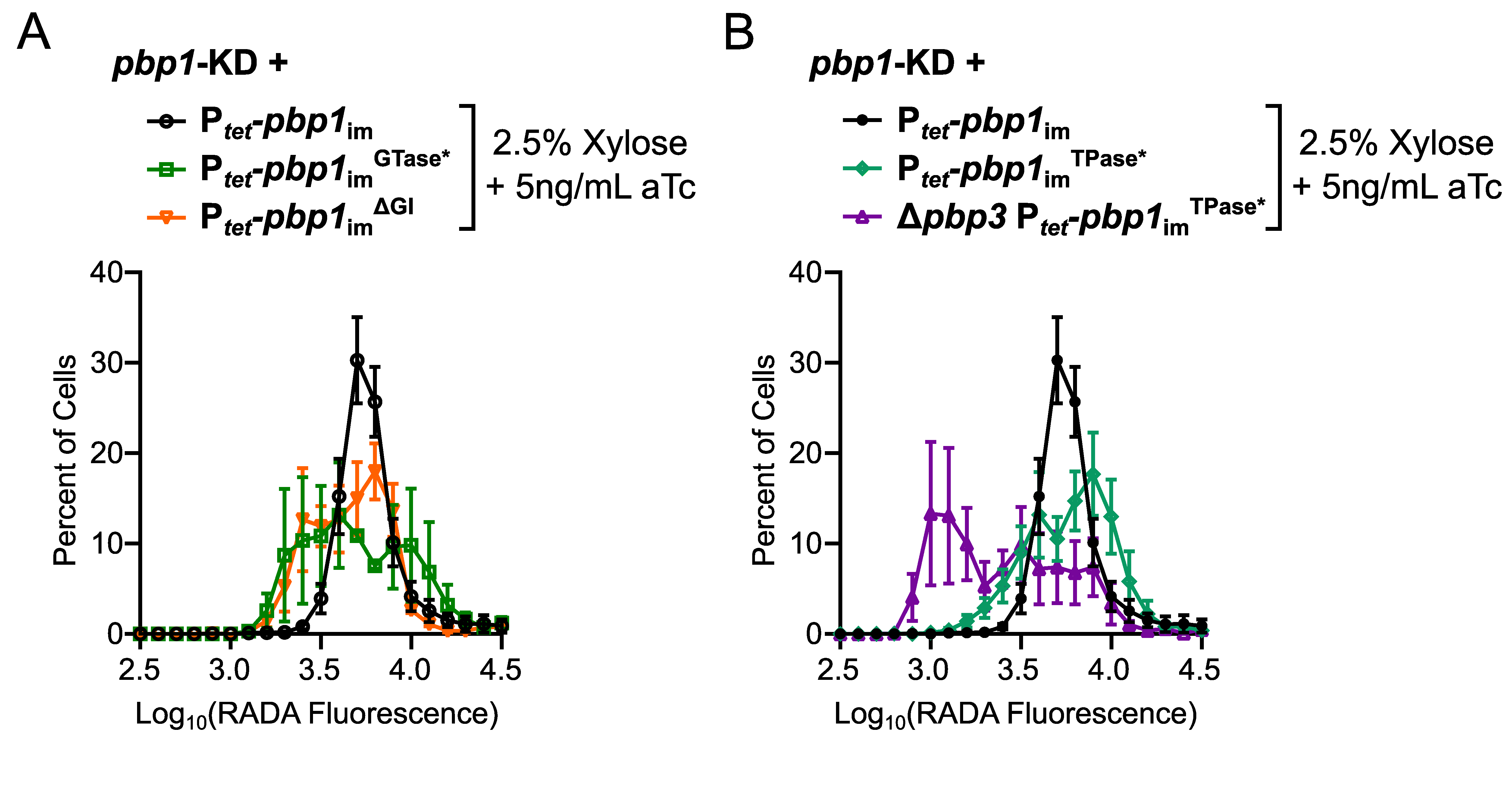

Supplement: S10 Fig — (A and B) C. difficile strains harboring the pbp1-KD cassette and pRPF185 plasmids encoding (A) Ptet-pbp1imGTase* (n = 3), Ptet-pbp1imΔGI (n = 3), or (B) Ptet-pbp1imTPase* in either the WT (n = 7) or Δpbp3 (n = 4) background were cultured in the presence of both 2.5% xylose and 5 ng/mL aTc as indicated in the scheme in Fig 2C to conditionally express the indicated pbp1 construct. The cells were then labeled with RADA for 10 min, fixed, and imaged by fluorescence microscopy. Data from Ptet-pbp1im (black circles) is the same data shown in Supplementary S2B Fig and reproduced here in both panels for the sake of comparison. The mean RADA fluorescence was quantified for each cell using SuperSegger. >690 cells were quantified across 3–7 independent experiments and symbols represent the mean and standard error of the mean across replicates. (TIF) [file pgen.1011746.s010.tif]

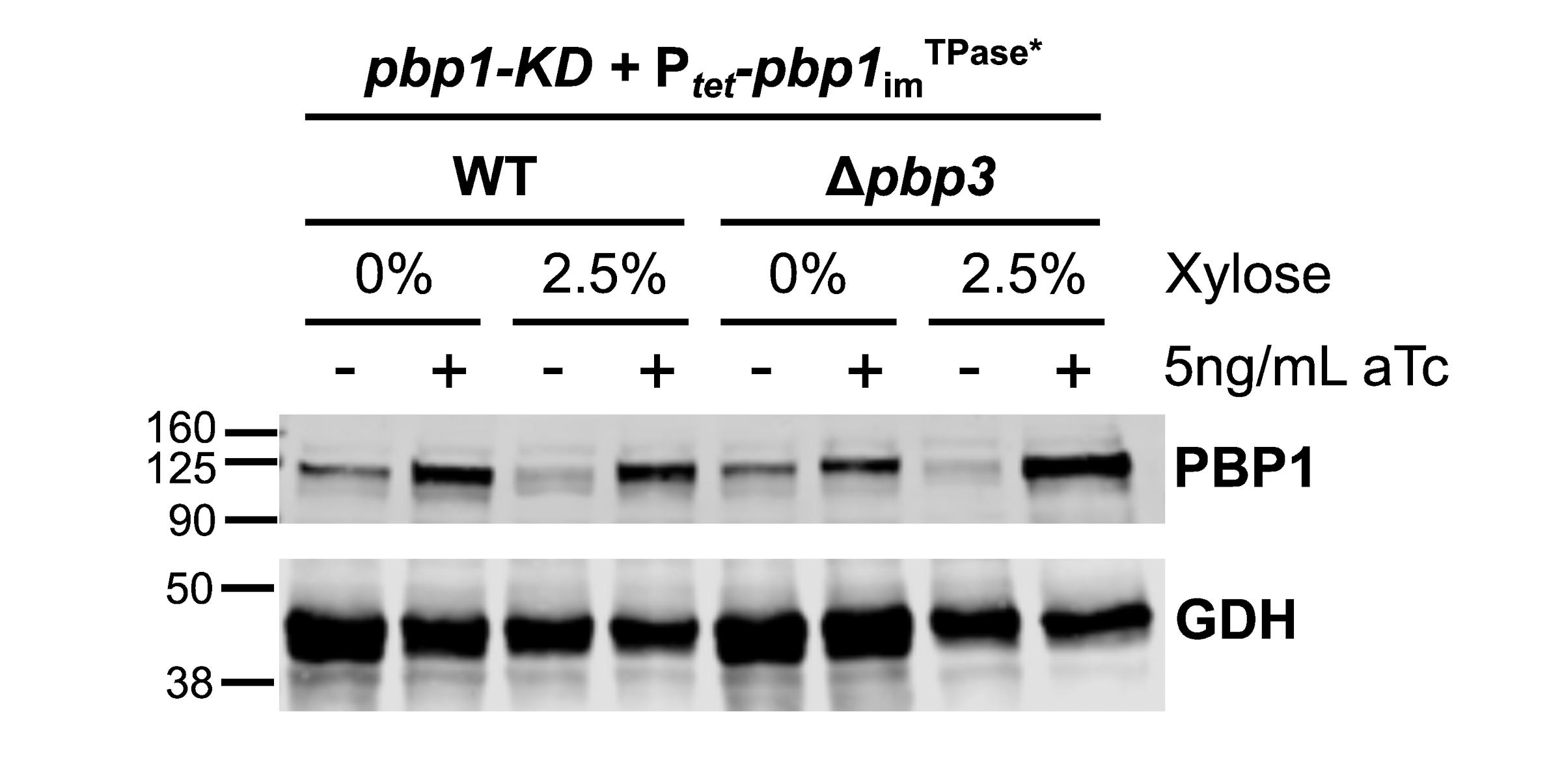

Supplement: S11 Fig — Western blot analyses of PBP1 in WT or Δpbp3 cells harboring pbp1-KD and pbp1TPase* complementation construct exposed to vehicle or 2.5% xylose and/or 5 ng/mL aTc per the scheme in Fig 2C. GDH (46.0 kDa) was used as a load control. Blots are representative of two independent experiments. (TIF) [file pgen.1011746.s011.tif]
